# A novel CAF population coordinates hyper-suppressive regulatory T cell recruitment and localization in lung cancer

**DOI:** 10.1038/s41590-026-02607-2

**Published:** 2026-08-11

**Authors:** Olivia R. Ringham, Monica Rivera, Lucas F. Loffredo, Melih Arda Ozsoy, Christina M. Healy, Maye F. Cheng, Yinuo Jin, Noah Chen, Kenia de los Santos-Alexis, Elham Azizi, Anjali Saqi, Matthew B. Buechler, Carla P. Concepcion-Crisol, Nicholas Arpaia

**Affiliations:** 1 Department of Microbiology & Immunology, Columbia University Irving Medical Center, New York, NY USA; 2 Herbert Irving Comprehensive Cancer Center, Columbia University Irving Medical Center, New York, NY USA; 3 Department of Immunology, University of Toronto, Toronto, Ontario Canada; 4 Department of Biomedical Engineering, Columbia University, New York, NY USA; 5 Irving Institute for Cancer Dynamics, Columbia University, New York, NY USA; 6 Department of Pathology and Cell Biology, Columbia University Irving Medical Center, New York, NY USA

**Keywords:** Tumour immunology, Translational immunology

## Abstract

Across many solid tumor types, cancer-associated fibroblasts (CAFs) are abundant and heterogeneous, with distinct subpopulations exerting immunomodulatory functions. Here we identify a novel population of immunomodulatory CAFs (imCAFs) in primary lung adenocarcinoma and pulmonary metastases, characterized by cell adhesion molecule L1-like (CHL1) expression and enriched in immune regulation and chemokine signaling programs. Through single-cell and spatial transcriptomics, we demonstrate that imCAFs are spatially colocalized with CXCR3^+^ regulatory T (T_reg_) cells, a hyper-suppressive subset accumulating at the tumor border. imCAFs produce CXCL9, driving CXCR3^+^ T_reg_ cell recruitment and promoting an immunosuppressive microenvironment. CXCR3^+^ T_reg_ cells display enhanced proliferative and suppressive capacity and are transcriptionally distinct from CXCR3^−^ counterparts. Genetic ablation of *Cxcr3* in T_reg_ cells or *Cxcl9* in stromal cells reduces T_reg_ cell accumulation, enhances CD8^+^ T cell activation and decreases tumor burden. Analogous CHL1^+^ imCAF-like fibroblasts in human non-small cell lung cancer colocalize with T_reg_ cells, and elevated *CHL1* expression is associated with reduced cytotoxicity and decreased progression-free survival, highlighting the imCAF–CXCL9–CXCR3^+^ T_reg_ axis as a promising therapeutic target.

## Abstract (web summary)

Ringham et al. identify immunomodulatory cancer-associated fibroblasts that influence regulatory T cell dynamics in lung cancer.

## Main

The tumor microenvironment (TME) is a highly complex multicellular network that plays a central role in dictating tumor progression and response to anticancer therapy. Solid tumors often exhibit desmoplastic features, which are characterized by the presence of activated CAFs. Rather than a uniform entity, CAFs encompass distinct subpopulations, with specialized roles in tumor progression, extracellular matrix remodeling and immune modulation^1,2^. Among these, immune-interacting CAFs represent a particularly important and functionally unique CAF niche found across various malagnancies^3^. In highly desmoplastic cancers, such as pancreatic ductal adenocarcinoma (PDAC), liver cancers and breast cancers, immune-interacting subpopulations of CAFs have been shown to regulate lymphocyte activation, recruitment and retention, and serve as key markers of prognosis and overall survival^4–7^. However, CAF subtypes and their impact on immune coordination in other solid tumors, such as lung adenocarcinoma (LUAD), remain less characterized.

Lung cancer remains the leading cause of cancer-related mortality in the United States, with adenocarcinoma as the most prevalent form^8^. Importantly, invasive LUAD commonly manifests in solid tumors that are poised to form a rich desmoplastic microenvironment. The lung is also among the most frequent sites of metastasis for many cancers, with up to half of metastatic colonization events forming solid tumors in this organ^9^. Evidence of stage-dependent immune cell infiltration in LUAD^10^ suggests that dynamic niches within the TME regulate antitumor immunity and influence tumor progression. Moreover, fibroblast niches within lung tumor stroma have been shown to express high levels of regulatory surface molecules, cytokines and chemokines that are canonically known to alter the functions of tumor-infiltrating lymphocytes^10–13^. Characterization of CAF populations in human lung cancers demonstrates that the presence and spatial distribution of distinct immune-modulating CAFs is a strong independent prognostic factor for patient survival^1,14^, which can influence the tumor–immune microenvironment and response to cancer treatments^15^. However, few studies have aimed to identify the ontogeny of distinct immune-interacting CAF populations or investigate the mechanisms by which CAFs modulate pro-tumor versus antitumor immune responses.

Although many leukocytes act to influence the immune landscape of lung tumors, T_reg_ cells play a particularly critical role in promoting tumor progression by dampening antitumor immunity^16^. Recently, several studies have suggested a role for distinct CAF populations in influencing T_reg_ cell dynamics and function in the TME of various solid tumor malignancies. In melanoma, breast and ovarian cancers, the presence of specific CAF subtypes has been correlated with enhanced T_reg_ cell trafficking, retention and activation in the TME, likely through chemokine signaling gradients and specialized receptor–ligand interactions^5,17,18^. In patients with LUAD, T_reg_ cells preferentially localize within the tumor stroma, and their increased presence correlates with significantly poorer prognosis^19^. However, the underlying mechanisms by which distinct CAF subsets modulate T_reg_ cell dynamics in lung tumors remain poorly understood.

In this report, we identify a population of imCAFs that play a pivotal role in shaping the lung TME by driving the recruitment of a highly immunosuppressive subset of CXCR3^+^ T_reg_ cells. Through the CXCL9–CXCR3 axis, imCAFs influence T_reg_ cell localization, activation and function, ultimately promoting an immunosuppressive TME and resulting in increased tumor burden in mouse models, with similar prognostic associations observed in humans. These results provide critical insights into the interplay between stromal and immune compartments in LUAD and suggest that targeting the imCAF–T_reg_ cell axis may represent a promising therapeutic avenue for improving antitumor immune responses in patients with lung cancer.

## Results

### imCAFs are enriched in lung tumor tissue

**Fig. 1:**
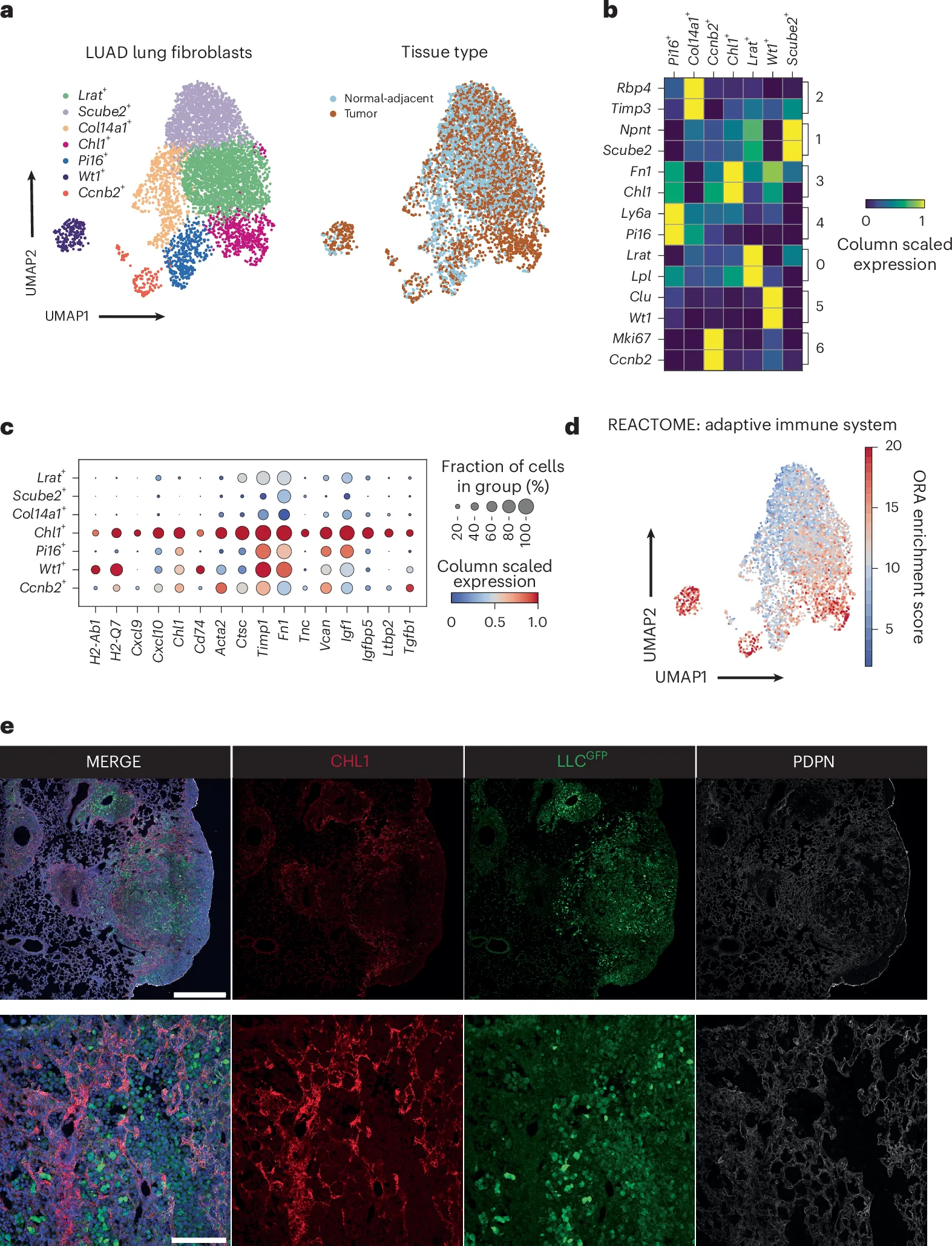
imCAFs are enriched in lung tumor tissue and express an immunomodulatory gene signature. **a**, Uniform manifold approximation and projection (UMAP) embedding of 5,616 fibroblast cells from LLC^GFP^ tumor-bearing lung lobes. Left: fibroblast cluster cell-type designation; right: tissue-type designation (*n* = 3 mice, 6 individually hashed tissue samples). **b**, Marker genes of fibroblast clusters. **c**, Dot plot of representative immunomodulatory genes from the top 100 DEGs enriched in the *Chl1*^+^ cluster. **d**, REACTOME pathway analysis for gene signatures associated with cells of the adaptive immune system in fibroblasts of LLC^GFP^ tumor-bearing lungs. **e**, Representative immunofluorescence microscopy images of LLC^GFP^ tumor-bearing lung lobes stained for CHL1 (red), LLC^GFP^ (green), podoplanin (PDPN; white) and DAPI (blue) derived from ten mouse lung lobes with similar results. Scale bars, 500 µm (upper images) and 200 µm (lower images).

To identify phenotypically and functionally distinct populations of CAFs within the lung TME, we intravenously injected mice with GFP-expressing Lewis lung carcinoma (LLC^GFP^) cells—an orthotopic model of LUAD—and performed single-cell RNA sequencing (scRNA-seq) on fluorescence-activated cell sorting (FACS)-sorted PDGFRα^+^ fibroblasts isolated from lung tumor tissue and normal-adjacent lung tissue (gating strategy in Supplementary Fig. 1a). Utilizing this transcriptomic approach, we uncovered seven transcriptionally distinct clusters of fibroblasts (Fig. 1a,b). We used hallmark genes to ascribe functional identities to most clusters, such as *Col14a1*^+^ fibroblasts^20^, *Scube2*^+^ alveolar fibroblasts^21^, *Pi16*^+^ adventitial fibroblasts^22^, *Wt1*^+^ mesothelial fibroblasts^23,24^, *Lrat*^+^ lipofibroblasts^25^ and a population of proliferating *Ccnb2*^+^ fibroblasts (Fig. 1b). Notably, one cluster—uniquely distinguished from all others by *Chl1* expression—did not express a known transcriptional signature of lung fibroblasts found at homeostasis or throughout other lung disease contexts. Intriguingly, *Chl1*^+^ fibroblasts were most predominantly detected (87.8% ± 6.7%) in tumor-associated tissue samples and largely absent in nonmalignant lung tissue, suggesting that this population represents bona fide CAFs.

Differential gene expression analysis revealed that this *Chl1*^+^ CAF cluster expresses an immunomodulatory gene signature—characterized by the expression of genes such as *Timp1*, *Cxcl9*, *Cxcl10*, *H2-Ab1*, *Tgfb1* and *Tnc* (Fig. 1c)—suggesting their potential role in dictating immune coordination in the TME. To infer the biological function of this cluster, we performed REACTOME analysis, which revealed that *Chl1*^+^ CAFs are uniquely enriched for pathways that facilitate interactions with cells of the adaptive immune system (Fig. 1d). Given their unique immunomodulatory transcriptional signature, predicted propensity to engage with adaptive immune cells and distinct enrichment within tumor-associated tissue, we termed this *Chl1*^+^ population ‘imCAFs’.

**Extended Data Fig. 1:**
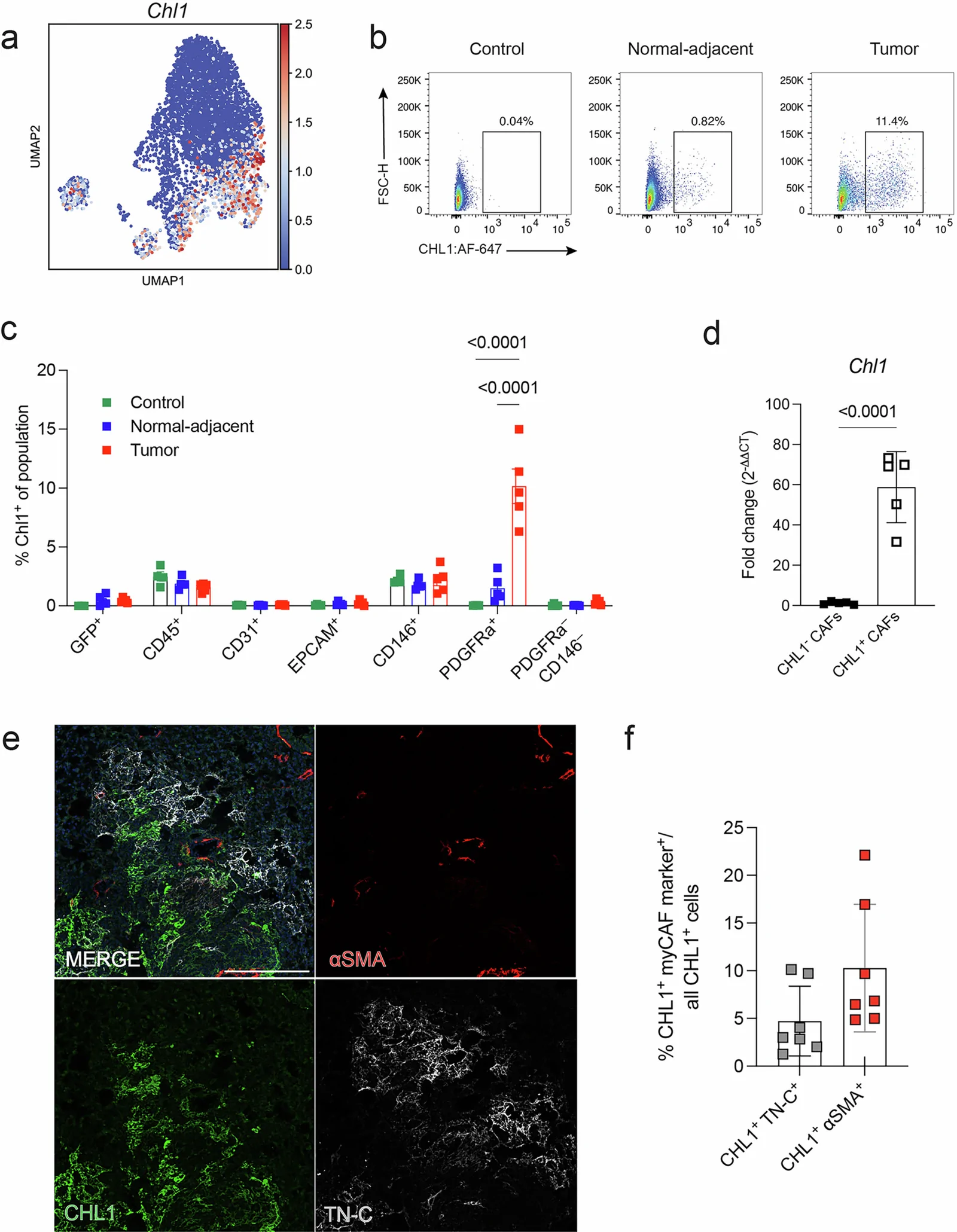
CHL1 expression is restricted to a subset of fibroblasts found within lung tumors. **a**, UMAP of CHL1 expression from fibroblasts found within LLC^GFP^ tumor-bearing lung lobes. **b**, Representative flow cytometry plots of CHL1 staining on PDGFRα^+^ fibroblasts from naïve control lungs, normal-adjacent tissue and lung tumor tissue. **c**, Quantification of CHL1 expression in various cell types found within naïve control lungs, normal-adjacent tissue, and lung tumor tissue. Representative data from 1 of 3 independent experiments (n=4 control, n=5 tumor-bearing). **d**, RT-qPCR of *Chl1* expression in CAFs FACS-sorted on CHL1 (n=5 biological replicates, see gating strategy in Extended Data Fig. 1). **e**, Representative immunofluorescence microscopy of tumor-bearing lung lobes stained for myCAF markers αSMA (red) and TN-C (white), and imCAF marker CHL1 (green) (n=7 biological replicates). Scale bar = 200μM. **f**, Frequency of myCAF marker^+^ imCAF^+^ cells relative to all imCAF^+^ cells. Statistical analysis done for flow cytometry data where three groups were compared using a one-way ANOVA, and RT-qPCR data where two groups are compared using two-tailed unpaired t tests. Mean and standard error displayed on graphs. Source data

As noted above, imCAFs are uniquely identified by high expression of cell adhesion molecule L1-like (*Chl1*; Fig. 1b and Extended Data Fig. 1a), which allowed for visualization by immunofluorescence microscopy and flow cytometric analysis and sorting using commercially available antibodies (Fig. 1e and Extended Data Fig. 1b–d). CHL1 is typically expressed in the brain and neurons and belongs to the L1 family of immunoglobulin neuronal cell adhesion molecules. Although *Chl1* expression has been previously observed in lung cancer^26^, to the best of our knowledge, its distinct cell-type-specific expression on LUAD-associated fibroblasts has not been demonstrated. Because imCAFs also express several genes associated with myofibroblastic CAFs (myCAFs), we next examined whether CHL1^+^ fibroblasts represent a distinct stromal population rather than a subpopulation of myCAFs. Co-staining tumor-bearing lung sections for the myCAF markers TN-C and αSMA together with the imCAF marker CHL1 revealed minimal overlap between these populations (Extended Data Fig. 1e,f), suggesting that imCAFs are distinct from canonical myCAF populations in the lung. Visualization of CHL1^+^ imCAFs by microscopy revealed that this population is localized primarily at the tumor border of LLC lung tumors (Fig. 1e).

**Extended Data Fig. 2:**
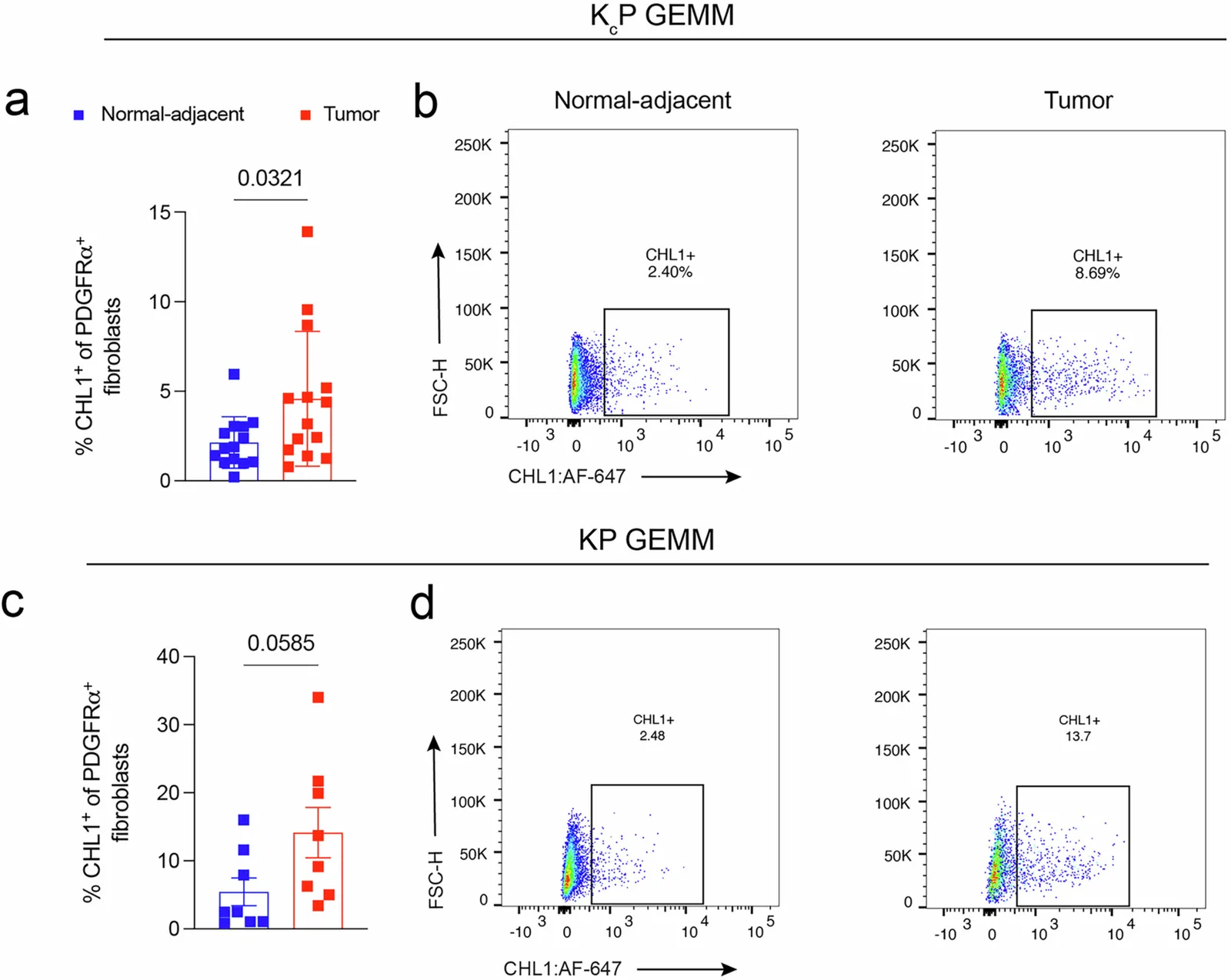
imCAFs are present across conditional Kras-driven GEMMs of lung cancer. **a,c** Frequency of CHL1^+^ fibroblasts in normal-adjacent or tumor tissue within tumor-bearing lung lobes in KP (n=14) (a) or K_c_P (n=8) (c)-driven models. **b, d**, Representative flow cytometry staining of CHL1 on PDGFRα^+^ cells found in normal-adjacent tissue and tumor tissue from tumor-bearing lung lobes. Statistical analysis done for flow cytometry data where two groups are compared using two-tailed unpaired t tests. Mean and standard error displayed on graphs. Source data

To determine whether imCAFs are also present in primary lung tumors that arise in situ, we next utilized two genetically engineered mouse models of LUAD: KP and K_c_P, which are driven by oncogenic *Kras*^*G12D*^ and *Kras*^*G12C*^ mutations, respectively, and are well-established and prevalent drivers of human LUAD^27^. Notably, CHL1^+^ fibroblasts were readily detected within tumors arising in both models (Extended Data Fig. 2a–d), demonstrating that imCAFs are not restricted to the LLC orthotopic model but are also present in spontaneous, genetically driven lung tumors. Taken together, these findings identify an immunomodulatory subset of CAFs—which we have termed ‘imCAFs’—that may serve as a critical stromal regulator of adaptive immune responses within the lung TME.

### imCAFs direct T_reg_ cell localization in lung tumors

**Fig. 2:**
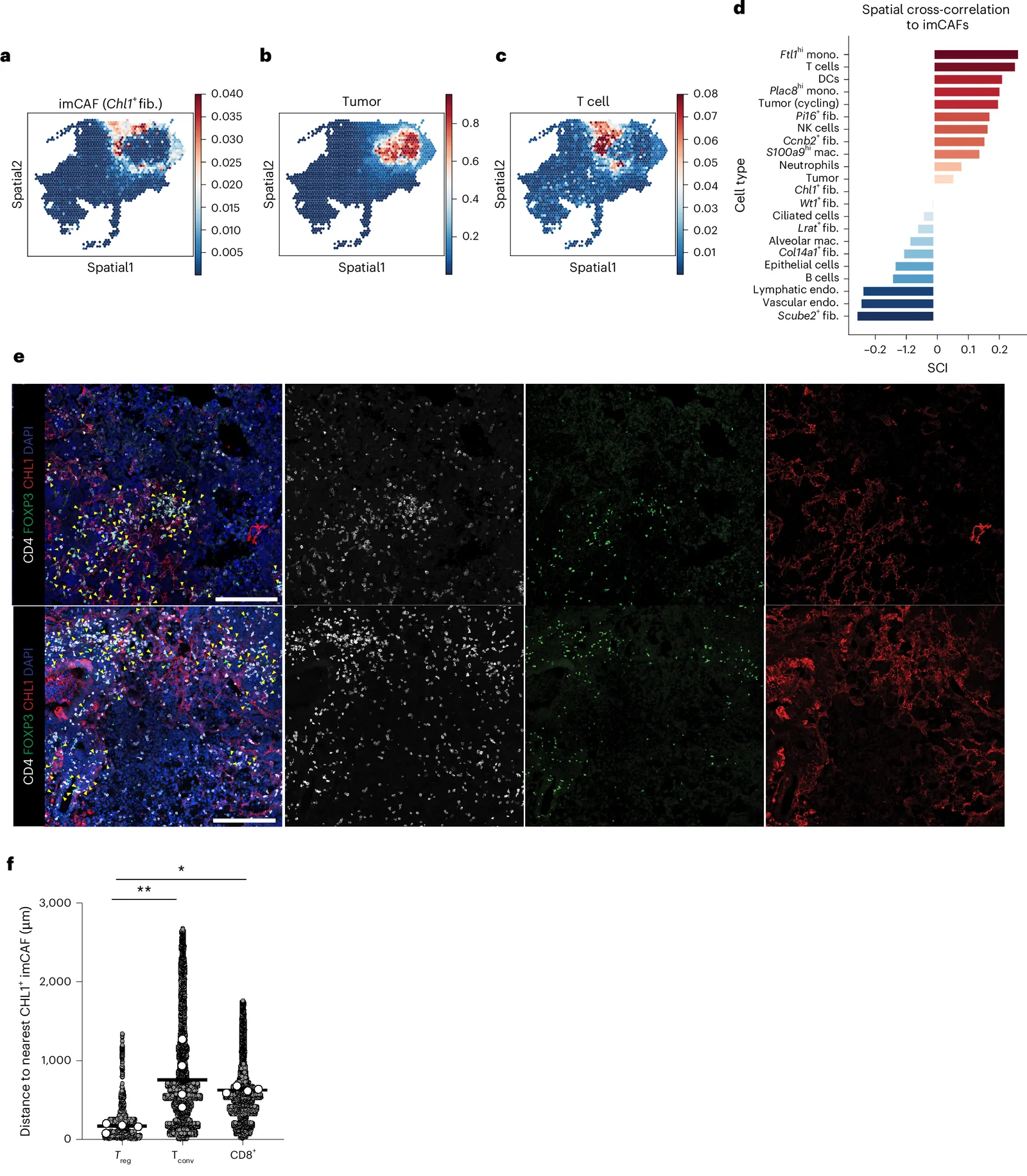
T_reg_ cells are predicted to interact with imCAFs in the lung TME and exhibit an imCAF-specific localization pattern. **a**–**c**, Spatial plots depicting estimated cell-type proportions (color intensity) of imCAFs (**a**), tumor cells (**b**) and T cells (**c**), using the cell2location mapping model. **d**, SCI of all cell types found within the concatenated scRNA-seq reference dataset of LLC^GFP^ tumor-bearing lungs and imCAFs. DCs, dendritic cells; endo., endothelium; fib., fibroblast; mac., macrophage; mono., monocyte; NK, natural killer. **e**, Representative immunofluorescence microscopy images of LLC^GFP^ lung tumors. Scale bars, 200 µm. Yellow arrowheads denote T_reg_ cells colocalized with CHL1^+^ imCAFs. **f**, *k*-means nearest-neighbor analysis of distance between a given CHL1^+^ imCAF and the closest T_reg_ cell (FOXP3^+^CD4^+^), T_conv_ cell (FOXP3^−^CD4^+^) and CD8^+^ T cell. White circles represent the mean distance of cells to imCAFs per lung tumor sampled (one tumor per mouse sampled on four individual mice); gray circles represent the distance of an individual cell to nearest imCAF. Statistical analysis performed using one-way analysis of variance (ANOVA) on mean values (white circles). T_reg_ versus T_conv_ (*P* = 0.0087), T_reg_ versus CD8 (*P* = 0.0298) *0.01 < *P* < 0.05, **0.001 < *P* < 0.01. Source data

**Extended Data Fig. 3:**
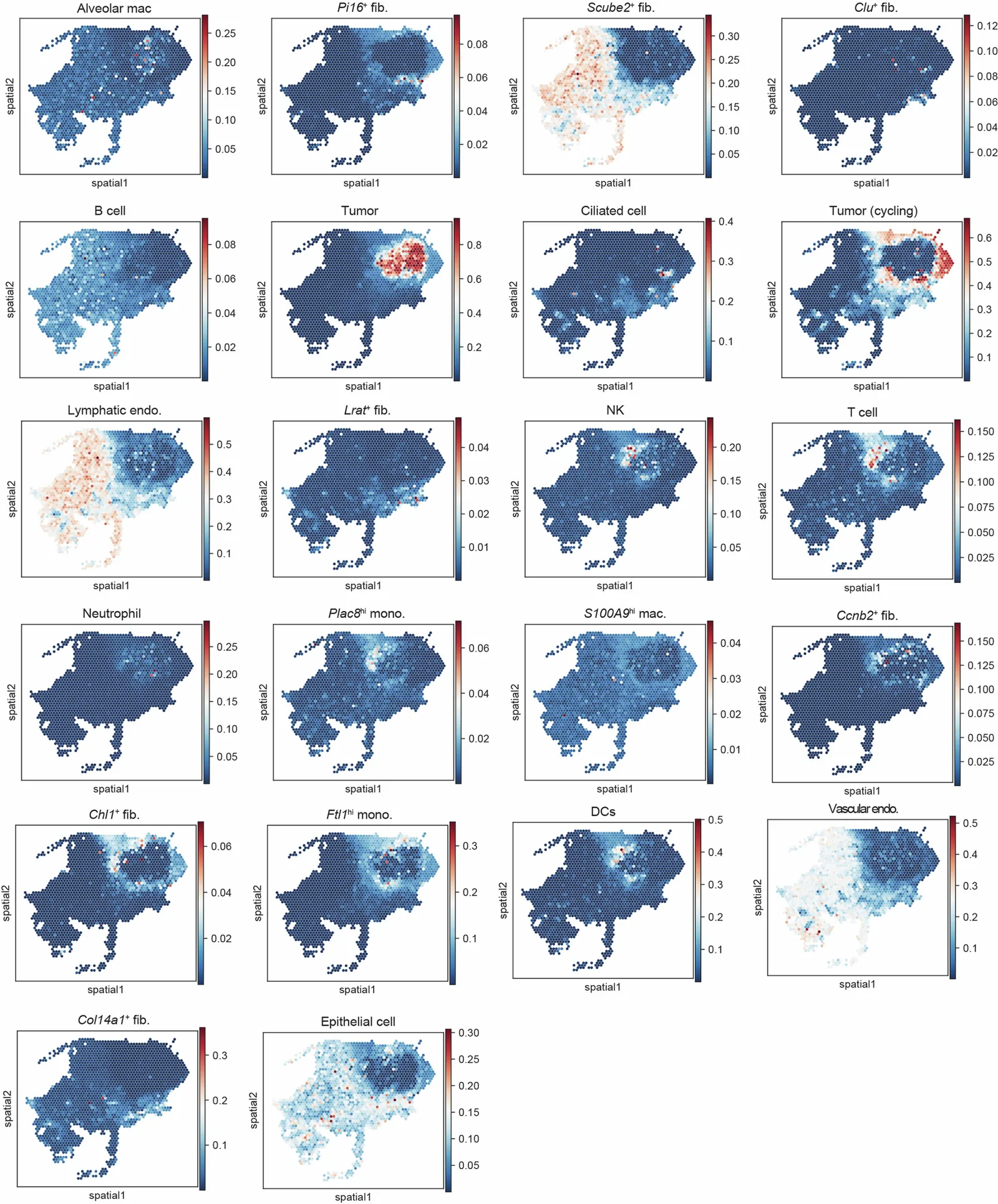
Spatial transcriptomic proportions of cell types within a tumor bearing lung lobe. Predicted proportions of cell types found within our reference dataset on a representative LLC^GFP^ tumor-bearing lung lobe mounted on a 10X Visium slide. Analysis was performed using the machine learning mapping model, Cell2Location, to map cell type-specific gene signatures from the reference dataset to Visium spots.

**Extended Data Fig. 4:**
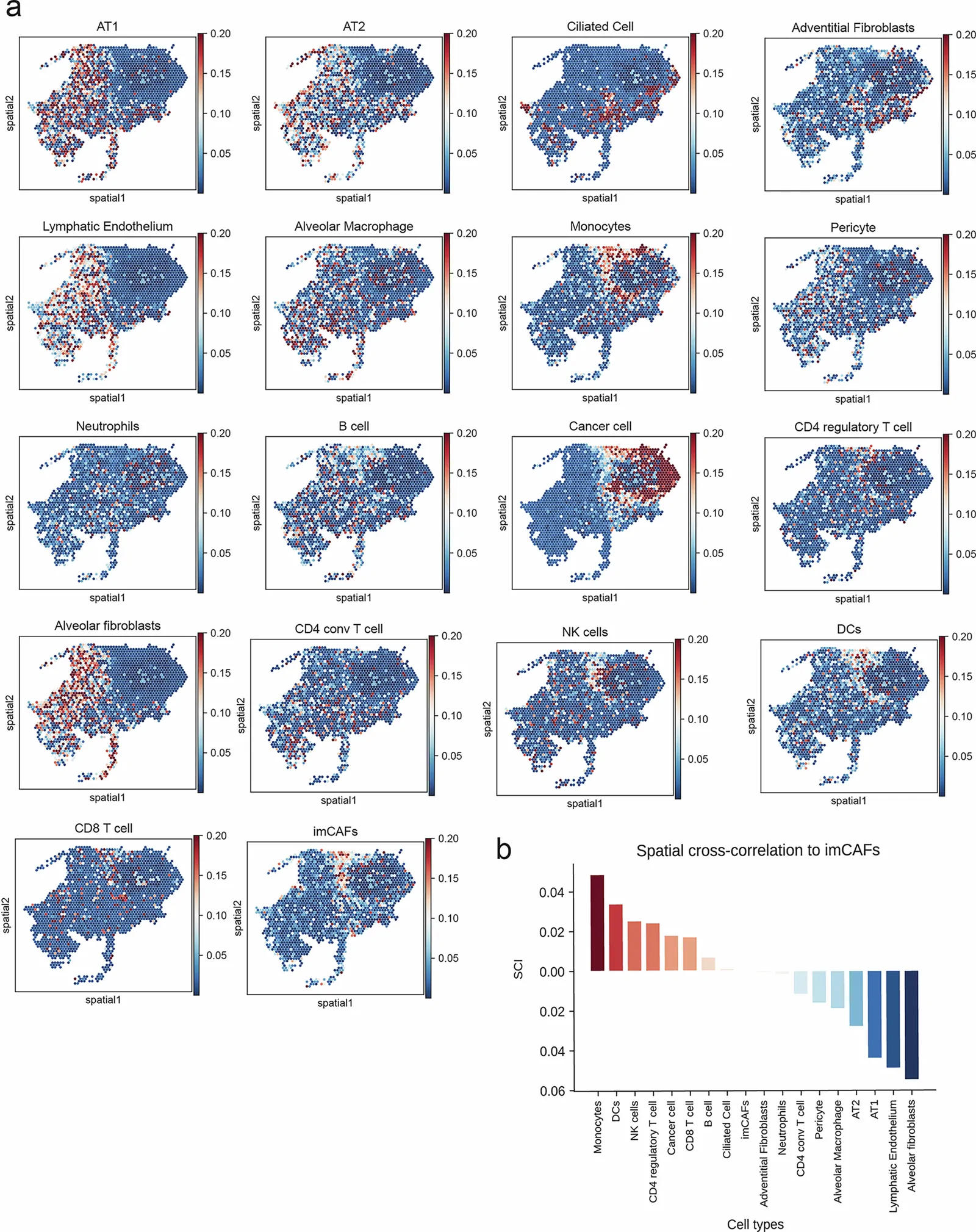
Spatial deconvolution predicts preferential Treg cell colocalization with imCAFs. **a**, Predicted proportions of cell types found within our reference dataset on a representative LLC^GFP^ tumor-bearing lung lobe mounted on a 10X Visium slide. Analysis was performed using Starfysh, a spatial deconvolution model, to map cell type-specific gene signatures from predefined gene sets (see Supplementary Data Table 1) to Visium spots. **b**, Spatial cross correlation of cell types found within tumor-bearing lung lobes based on spatial deconvolution analysis utilizing Starfysh.

To understand how imCAFs interact with various cell types in the TME, we performed spatial transcriptomic analysis. This approach enabled us to map cell-type-specific gene signatures to distinct regions of the lung TME tissue architecture, allowing for an unbiased analysis of cell–cell interactions while considering both spatial and transcriptional contexts. To this end, we generated an scRNA-seq dataset of all cells found within LLC tumor-bearing lungs, performed integration with our aforementioned fibroblast dataset and utilized a machine learning mapping model to map transcriptionally distinct clusters of cells onto the spatial transcriptomics slide (Extended Data Fig. 3). As observed by immunofluorescence microscopy, imCAFs appeared to be highly colocalized to the peritumoral area of lung tumors (Fig. 2a,b). In line with our results from REACTOME pathway analysis, T cells were also enriched in this region (Fig. 2c) and exhibited a high spatial cross-correlation index (SCI) relative to imCAFs (Fig. 2d), suggesting T cells and imCAFs may engage in specialized interactions. Given that our reference dataset did not possess high enough resolution to differentiate T cell subsets in the TME, we utilized immunofluorescence microscopy to systematically evaluate if specific T cell subsets exhibit unique colocalization with imCAFs (Fig. 2e). Strikingly, as compared to conventional CD4^+^ T (T_conv_) cells and CD8^+^ T cells, FOXP3^+^ T_reg_ cells appeared to be highly colocalized to CHL1^+^ cells in lung tumors by *k*-means nearest-neighbor analysis (Fig. 2f), suggesting their unique propensity to interact with imCAFs within the TME. Consistent with our findings from imaging experiments, deconvolution of spatial transcriptomic data to model localization of distinct T cell subsets utilizing Starfysh (Extended Data Fig. 4a and Supplementary Table 1)—a reference-free, deep generative modeling tool—demonstrated T_reg_ cells as having the highest SCI index of the T cell subsets (Extended Data Fig. 4b).

**Extended Data Fig. 5:**
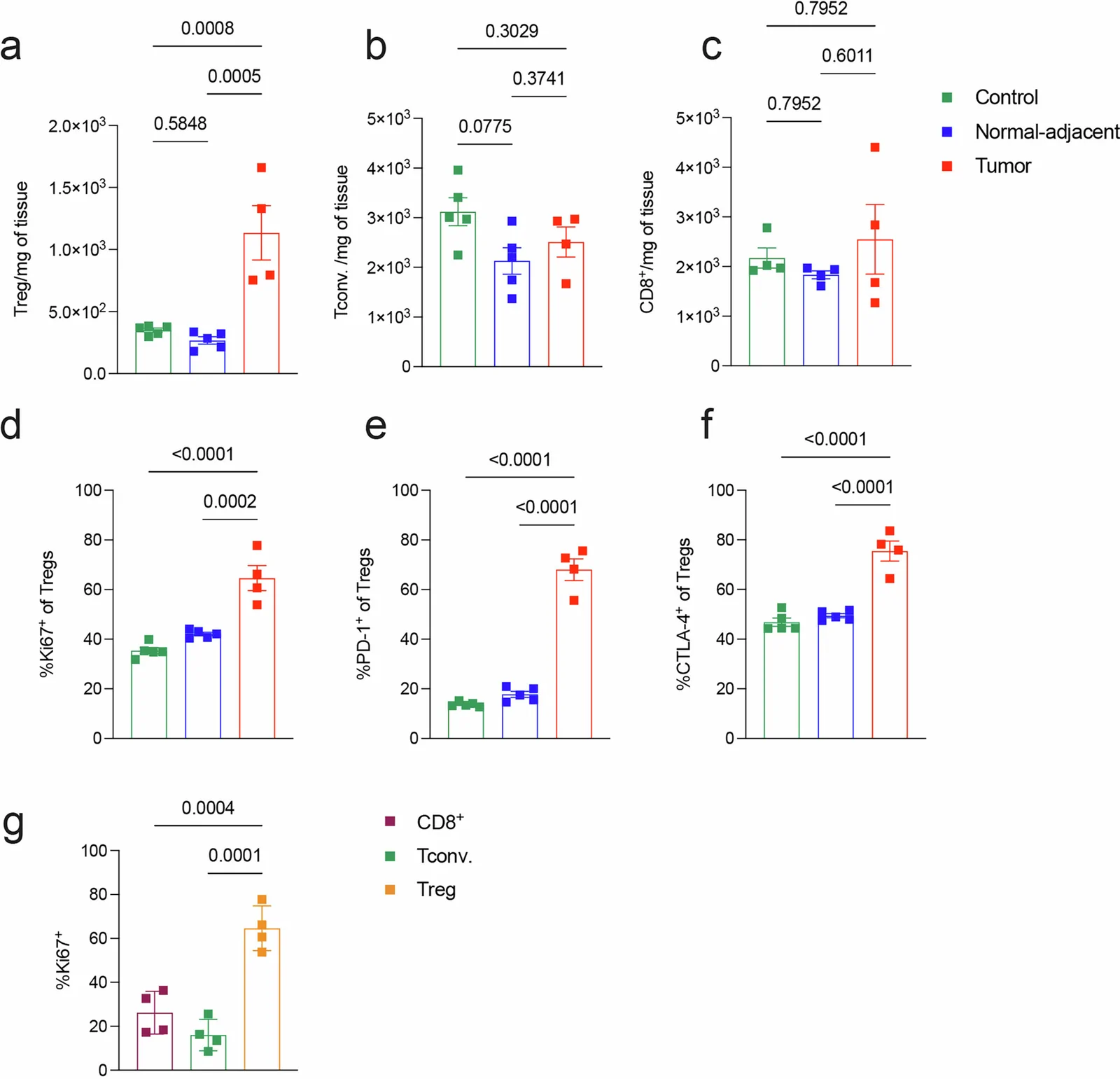
Treg cells are uniquely abundant in lung tumor tissue and upregulate functional markers. **a**, Treg cells, **b**, Tconv. cells, and **c**, CD8^+^ T cells per mg of tissue in splenic, normal-adjacent and lung tumor tissue. **d**, Frequency of Ki67^+^, **e**, PD-1^+^, and **f**, CTLA4^+^ Treg cells in splenic, normal-adjacent and lung tumor tissue (n=4 control, n=5 normal-adjacent tissue, n=4 tumor tissue). **g**, Frequency of Ki67^+^ cells across Treg cells, Tconv. cells and CD8^+^ T cells in lung tumor tissue (n=4). Statistical analysis done for flow cytometry data where three groups were compared using one-way ANOVA. Mean and standard error displayed on graphs. Source data

Examination of the T cell compartment in tumor-bearing lungs revealed that T_reg_ cells were found to be uniquely abundant in lung tumor tissue relative to normal-adjacent lung tissue and control lungs that lack LLC tumors (Extended Data Fig. 5a). This was not observed for conventional CD4^+^ or CD8^+^ T cell subsets, for which there were no differences in abundance across lung tissue types (Extended Data Fig. 5b,c). Moreover, relative to T_reg_ cells from normal-adjacent and control lung tissue, a significantly greater frequency of tumor T_reg_ cells expressed functional markers of proliferation and immunosuppression (Extended Data Fig. 5d–f), with tumor T_reg_ cells also being the most proliferative among all T cell subsets (Extended Data Fig. 5g). Taken together, the strong spatial association between imCAFs and T_reg_ cells, along with the distinct activation phenotype of lung tumor T_reg_ cells relative to non-tumor-associated T_reg_ cells, suggests imCAFs may play a role in shaping the local immune landscape through preferential interactions with T_reg_ cells within the TME.

### imCAF-derived CXCL9 drives T_reg_ cell recruitment to the lung TME

**Fig. 3:**
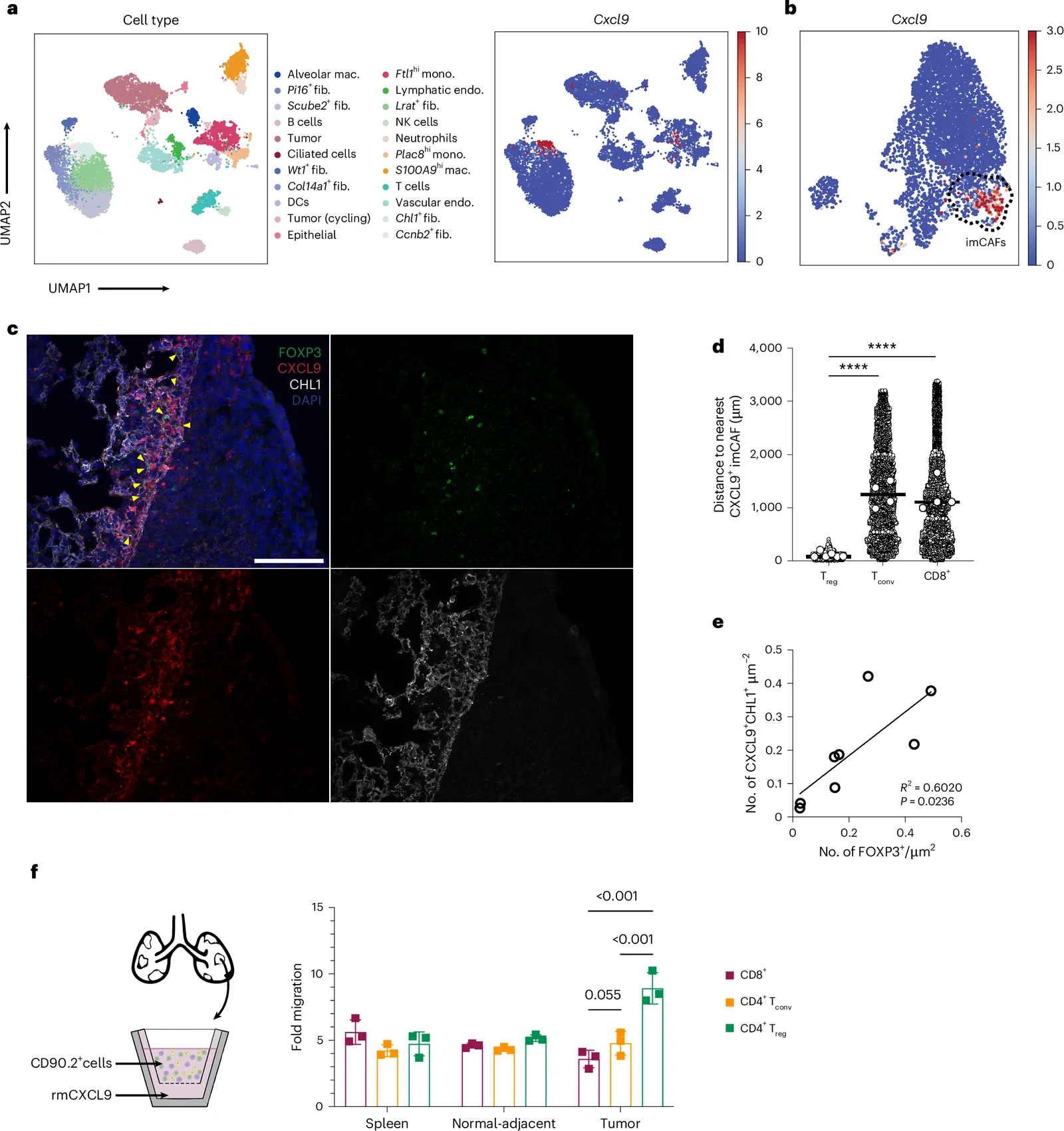
imCAF-derived CXCL9 drives tumor T_reg_ cell recruitment to the lung TME. **a**, UMAP of cell types found within an LLC^GFP^ tumor-bearing lung lobe in our combined fibroblast/reference dataset (left). *Cxcl9* gene expression within an LLC^GFP^ tumor-bearing lung lobe (right). **b**, UMAP of *Cxcl9* expression in fibroblast-enriched scRNA-seq dataset. **c**, Representative immunofluorescence microscopy image of LLC^GFP^ tumors stained for CXCL9 (red), FOXP3 (green), CHL1 (white) and DAPI (blue). Arrowheads point to FOXP3^+^ cells localized to CXCL9^+^CHL1^+^ imCAFs. Scale bar, 100 µm. **d**, *k*-means nearest-neighbor analysis of distance between a given CHL1^+^CXCL9^+^ imCAF and the closest T_reg_ cell (FOXP3^+^CD4^+^), T_conv_ cell (FOXP3^−^CD4^+^) and CD8^+^ T cell (*n* = 4 biological replicates). T_reg_ versus T_conv_ (*P* = 8.68 × 10^−^^6^), T_reg_ versus CD8^+^ (*P* = 8.68 × 10^−6^). **e**, Pearson correlation between number of T_reg_ cells and number of CXCL9-expressing imCAF cells per area of lung tumor. **f**, Schematic of experimental setup for competitive Transwell chemotaxis assay toward a CXCL9 gradient using splenic, normal-adjacent or tumor-derived T cells (left). rmCXCL9, recombinant mouse CXCL9. Quantification of change in fold migration of T cell subsets relative to no insert control of cells from a given tissue (right). Data are representative of three independent experiments (*n* = 3 replicates per condition; tumor-derived T_reg_ versus T_conv_ (*P* = 2.75 × 10^−6^), tumor-derived T_reg_ versus CD8^+^ (1.10 × 10^−7^)). Statistical analysis was performed for data where three groups were compared using a one-way ANOVA. The mean and standard error are displayed on the graphs. ****P < 0.0001. Source data

**Extended Data Fig. 6:**
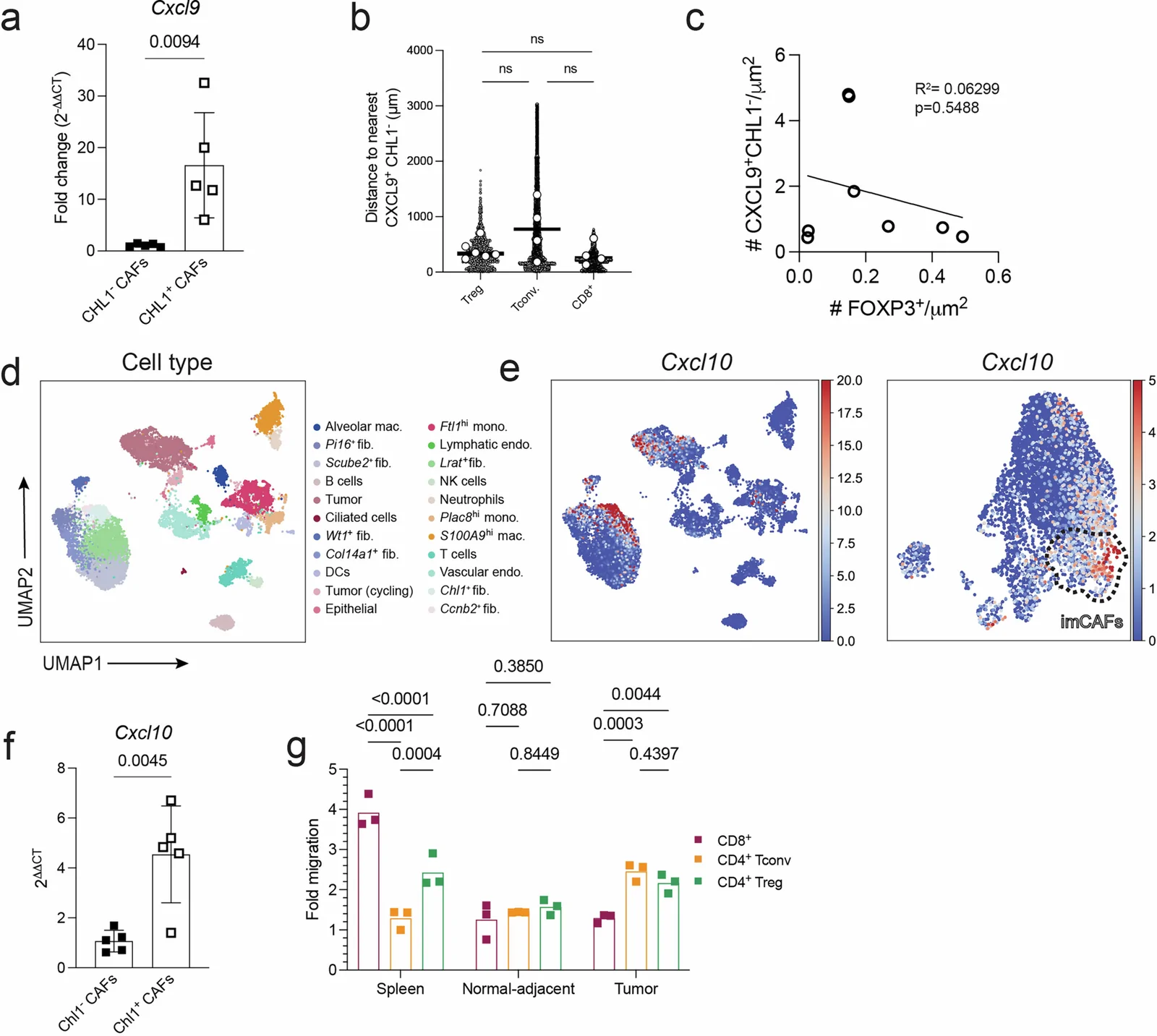
imCAF-derived CXCL9 drives Treg cell migration to the TME. **a**, RT-qPCR for *Cxcl9* in CHL1^−^ and CHL1^+^ CAFs (n = 5 mice). **b**, K-means nearest neighbor analysis of CXCL9^+^ CHL1^−^ cells and the nearest Treg cell, Tconv. cell, or CD8^+^ T cell in a given lung tumor (n=6 mice Treg; n=4 mice Tconv., n=4 mice CD8^+^) **c**, Pearson correlation between number of Treg cells and number of CXCL9^+^ CHL1^−^ cells per area of lung tumor. **d**, UMAP of cell types found within a LLC^GFP^ tumor-bearing lung lobe in our combined fibroblast/ reference dataset (left). *Cxcl10* gene expression within a LLC^GFP^ tumor-bearing lung lobe (right). **e**, UMAP of *Cxcl10* expression in fibroblast enriched scRNAseq dataset. **f**, RT-qPCR of *Cxcl10* expression in CAFs FACS-sorted on CHL1 (see gating strategy in Supplementary Fig. 1). **g**, Quantification of change in fold migration of T cell subsets relative to no insert control of cells from a given tissue to recombinant mCXCL10. Representative data of 3 independent experiments (n = 3 replicates per condition). Statistical analysis done for RT-qPCR data where two groups are compared using two-tailed unpaired t tests, and for comparing k-means nearest neighbor distances between three groups using a one-way ANOVA. Mean and standard deviation displayed on graphs; ns: not significant, *:0.01<p<0.05, **:0.001<p<0.01 Source data

Among the top differentially expressed genes (DEGs) in the imCAF population was *Cxcl9*, a well-established T cell chemoattractant^28^. *Cxcr3*-expressing T cells are drawn to CXCL9 gradients, and this axis is critical in lymph nodes where fibroblastic reticular cells in the T cell zone secrete CXCL9 to facilitate T cell migration and maturation^29–31^. In tumor-bearing lung lobes, imCAFs were found to be the most prominent expressors of *Cxcl9* relative to all other cell types (Fig. 3a,b), including dendritic cells and macrophages, which are known to highly express CXCR3 ligands such as CXCL9 (refs. ^32,33^). Increased *Cxcl9* expression by CHL1^+^ imCAFs compared to CHL1^−^ CAFs was confirmed by quantitative PCR with reverse transcription (RT–qPCR; Extended Data Fig. 6a). Notably, T_reg_ cells were found to preferentially localize to CXCL9-producing imCAFs relative to other T cell subsets, and the abundance of CXCL9^+^ imCAFs correlated with the number of T_reg_ cells found within a given lung tumor (Fig. 3c–e). Importantly, T_reg_ cells did not demonstrate preferential localization to CXCL9^+^CHL1^−^ cells, nor did T_reg_ cell abundance correlate with the abundance of CXCL9^+^CHL1^−^ cells (Extended Data Fig. 6b,c), demonstrating that non-imCAF sources of CXCL9 do not affect T_reg_ cell localization or recruitment to the TME. Additionally, utilizing a competitive Transwell migration assay, T_reg_ cells harvested from lung tumor tissue migrated toward CXCL9 gradients to a significantly greater extent than other tumor T cell subsets (CD4^+^ T_conv_ and CD8^+^ T cells) and T cells isolated from normal-adjacent lung tissue or spleen (Fig. 3f). Notably, *Cxcl10*, encoding another CXCR3 ligand, was also among the genes upregulated in imCAFs in our scRNA-seq dataset, and its elevated expression in CHL1^+^ imCAFs was confirmed by RT–qPCR (Extended Data Fig. 6d–f). However, in contrast to CXCL9, CXCL10 gradients did not preferentially induce migration of tumor-infiltrating T_reg_ cells (Extended Data Fig. 6g). These findings suggest that tumor-infiltrating T_reg_ cells are preferentially responsive to CXCL9, and that CXCL9-producing imCAFs may be critical in driving T_reg_ cell migration and accumulation within the lung TME.

**Extended Data Fig. 7:**
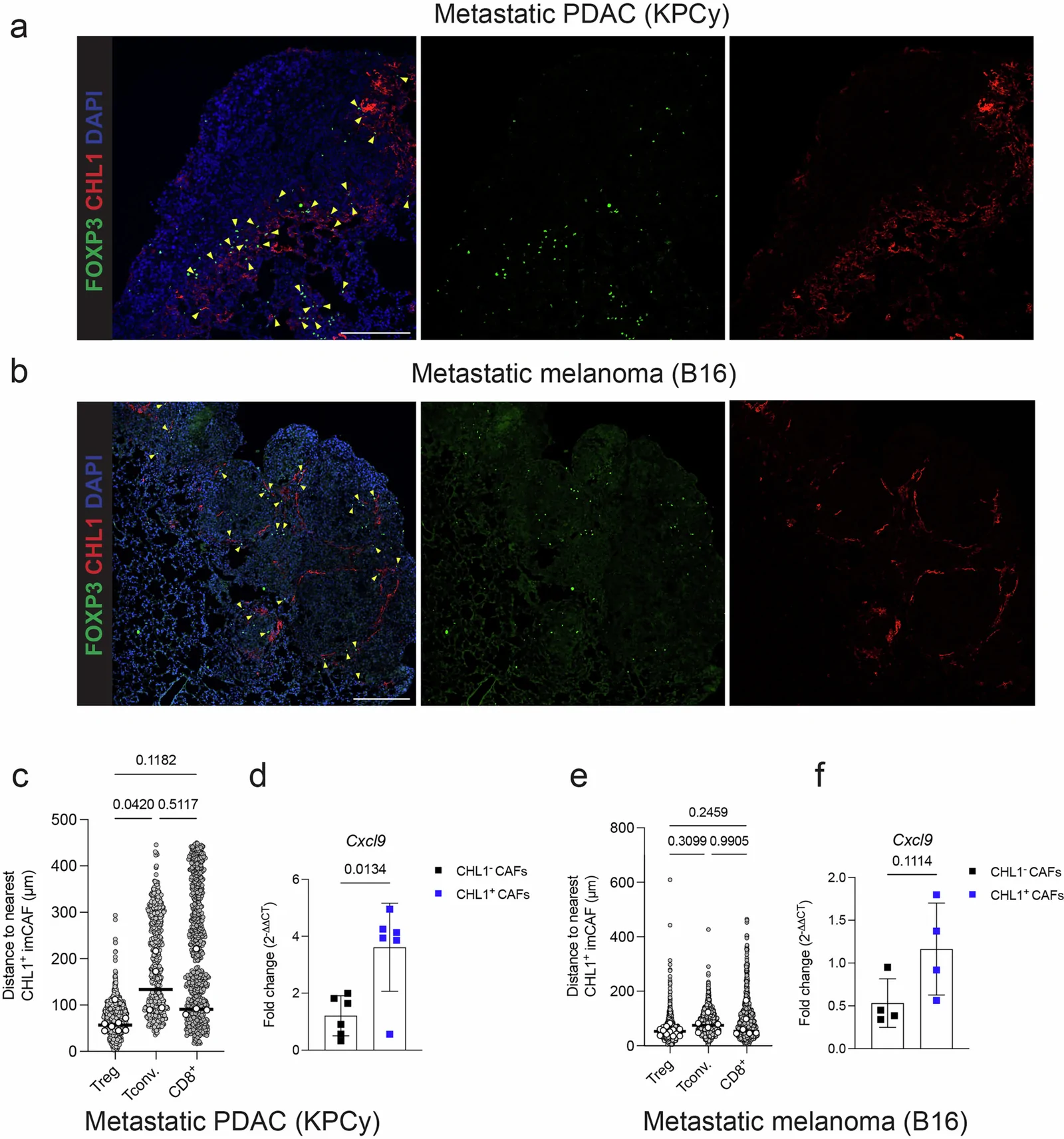
Treg cell localization to CXCL9-producing imCAFs is conserved across lung-metastatic cancer models. **a-b**, Representative immunofluorescence microscopy images of (**a**) metastatic PDAC (KPCy) and (**b**) metastatic melanoma (B16F10) lung tumors stained for FOXP3 (green), CHL1 (red), and DAPI (blue). Yellow arrows point to FOXP3^+^ Treg cells. **c, e**, K-means nearest neighbor analysis of distance between a given CHL1^+^ imCAF and the closest Treg cell (FOXP3^+^ CD4^+^) Tconv. cell (FOXP3^−^ CD4^+^) and CD8^+^ T cell in in KPCy and B16F10 lung tumors, respectively (n = 4 mice for KPCy; n = 6 mice for B16 – matched imaging FOXP3 + CD4 and FOXP3 + CD8 staining for each mouse tumor analzyed). **d, f**, RT-qPCR for *Cxcl9* in CHL1^−^ and CHL1^+^ CAFs in KPCy (n = 6 mice) and B16F10 lung tumors (n = 4 mice), respectively. Statistical analysis done for RT-qPCR data where two groups are compared using two-tailed unpaired t tests, and for comparing k-means nearest neighbor distances between three groups using a one-way ANOVA. Mean and standard deviation displayed on graphs; ns: not significant, *:0.01<p<0.05, **:0.001<p<0.01. Source data

The lung is a critical and frequent site of metastasis for various types of cancer due to its unique vascular and physiological characteristics^34^. Thus, we reasoned that imCAFs may exist in metastatic lung lesions that arise from other cancer types. To evaluate if imCAFs are present in other models of metastatic malignancy in the lung, we utilized the B16F10 and KPCy cell lines to model metastatic melanoma and PDAC metastasis, respectively. CHL1^+^ imCAFs were present within lung tumor tissue of both models and were found to exhibit a peritumoral localization pattern (Extended Data Fig. 7a,b), similar to that observed in the LLC model. Interestingly, preferential colocalization of T_reg_ cells to imCAFs was only observed in the KPCy model, in which imCAFs upregulate expression of *Cxcl9* (Extended Data Fig. 7c,d). In B16F10 lung metastases, T_reg_ cells did not demonstrate preferential colocalization to imCAFs relative to other T cell subsets, which may be attributed to the lack of significant *Cxcl9* expression by imCAFs relative to CHL1^−^ CAFs in this model (Extended Data Fig. 7e,f). These findings suggest that imCAFs are a common feature of primary lung tumors and metastatic lung lesions across other cancer models, with their ability to recruit T_reg_ cells being influenced by context-dependent *Cxcl9* expression.

### CXCR3 identifies a hyper-suppressive T_reg_ cell subset

**Fig. 4:**
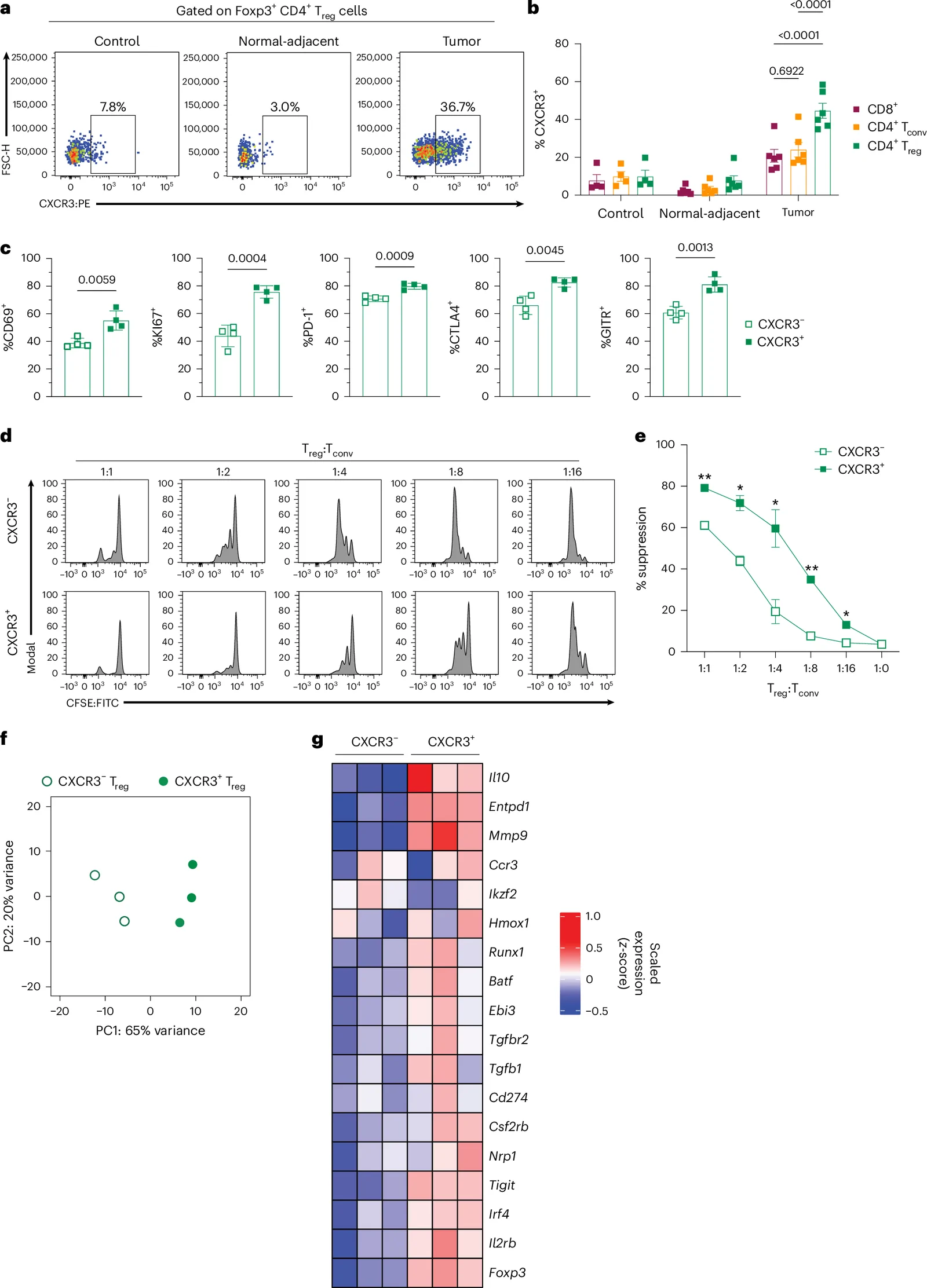
CXCR3^+^ T_reg_ cells are a distinct, hyper-immunosuppressive subset in lung tumors. **a**, Representative flow cytometry staining of CXCR3 on T_reg_ cells found in naive control lung tissue as well as normal-adjacent tissue and tumor tissue from LLC^GFP^ tumor-bearing lungs. **b**, Frequency of CXCR3^+^ cells in different T cell subsets from control, normal-adjacent and tumor tissues. Combined data from two independent experiments (*n* = 4 control, *n* = 6 tumor-bearing; tumor-derived T_reg_ versus T_conv_ (*P* = 8.68 × 10^−6^), tumor-derived T_reg_ versus CD8^+^ (*P* = 8.68 × 10^−6^)). **c**, Quantification of flow cytometry staining for functional markers in CXCR3^−^ or CXCR3^+^ tumor tissue-derived T_reg_ cells. Data are representative of two independent experiments (*n* = 4). **d**, Representative CFSE labeling of naive T_conv_ cells after co-incubation with various concentrations of either CXCR3^−^ or CXCR3^+^ splenic T_reg_ cells in a T_reg_ cell suppression assay. **e**, Quantification of the frequency of CFSE^+^ T_conv_ cells (right-most peak in **d**) after co-incubation with varying proportions of T_reg_ cells from *Foxp3*^GFP^ male mice (*n* = 3 replicates per condition). Data are representative of two independent experiments. *P* values (left to right): *P* = 0.0045, *P* = 0.01, *P* = 0.03, *P* = 0.001, *P* = 0.01. **f**, PCA of CXCR3^−^ and CXCR3^+^ T_reg_ cells from lung tumor tissue from *Foxp3*^GFP^ male mice (*n* = 3 mice). **g**, Heat map of hyper-suppressive and effector-like gene signatures enriched in CXCR3^+^ T_reg_ cells versus CXCR3^−^ T_reg_ cells. Statistical analysis was performed for flow cytometry data and T_reg_ cell suppression data where two groups were compared using two-tailed unpaired *t*-tests, and for flow cytometry data where three groups were compared using one-way ANOVA. The mean and standard error are displayed on the graphs; *0.01 < *P* < 0.05, **0.001 < *P* < 0.01. Source data

**Extended Data Fig. 8:**
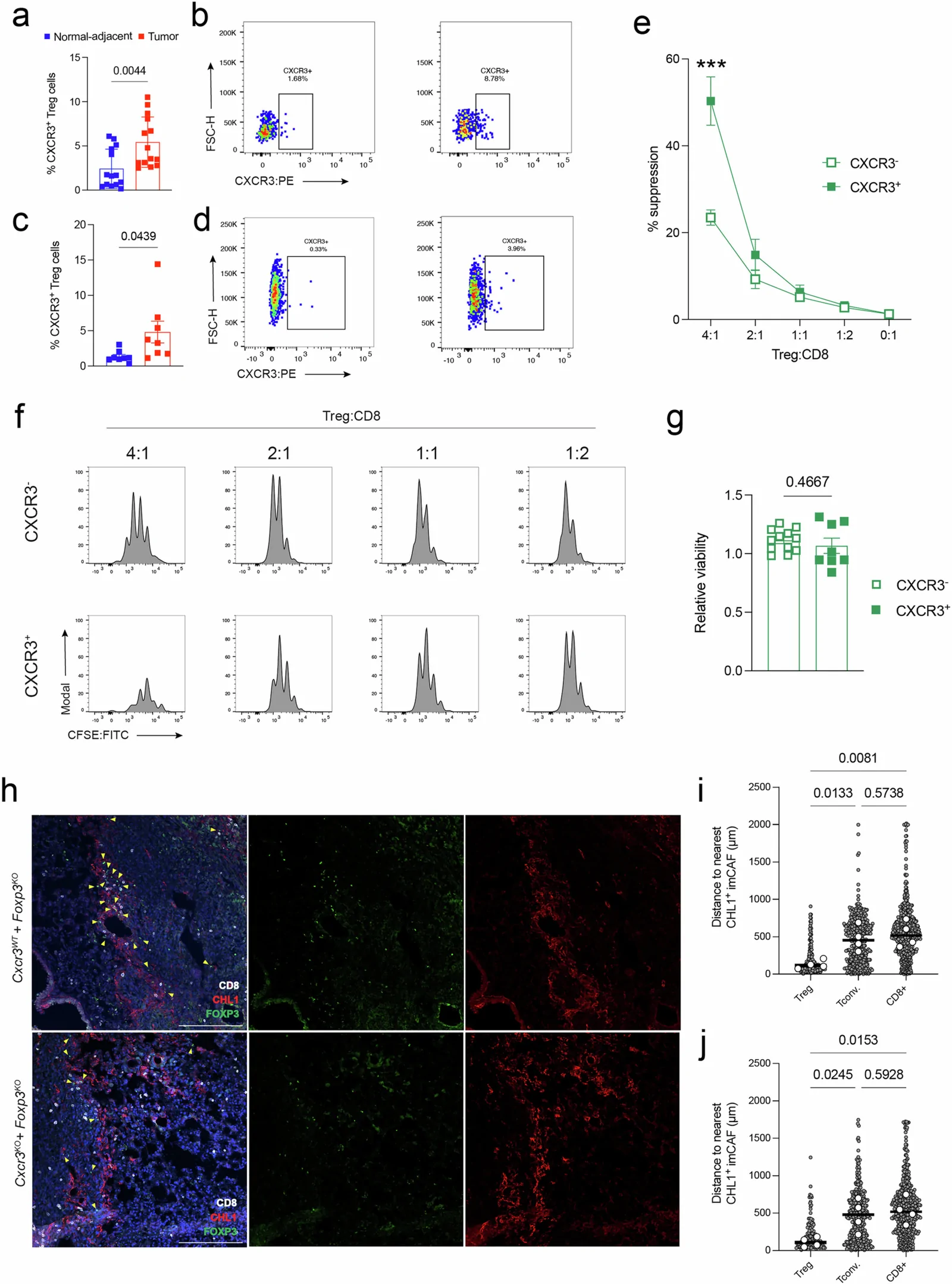
CXCR3+ Tregs display enhanced suppressive activity while Treg expression of CXCR3 is dispensable for direct inhibition of CTL effector function and Treg spatial association with imCAFs. **a, c**, Frequency of CXCR3^+^ Treg cells found in normal-adjacent or tumor tissue within tumor-bearing lung lobes in KP (n=14) (A) or K_c_P (n=8) (C)-driven models. **b, d**, Representative flow cytometry staining of CXCR3 on FOXP3^+^ Treg cells found in normal-adjacent tissue and tumor tissue from tumor-bearing lung lobes in KP (B) or K_c_P (D)-driven models. **e**, Quantification of frequency of CFSE^+^ CD8^+^ cells (right-most peak) after coincubation with varying proportions of Treg cells *Foxp3*^*GFP*^ male mice (n = 3 replicates/condition, p=0.0008). **f**, Representative CFSE labeling of CD8^+^ cells after coincubation with various concentrations of either CXCR3^−^ or CXCR3^+^ splenic Treg cells in a Treg cell suppression assay. **g**, Quantification of relative viability of LLC^OVA-LUC^ cells from CTL killing assay with Treg cell subsets. Combined data from 2 independent experiments (CXCR3^−^ n=12, CXCR3^+^ n=8). **h**, Representative immunofluorescence microscopy images of LLC^GFP^ lung tumors from mixed bone marrow chimera mice in respective groups. CD8 = white, FOXP3 = green, CHL1 = red, DAPI = blue. Yellow arrows delineate Treg cells colocalized to imCAFs. Scale bar = 200μM. **i-j**, K-means nearest-neighbor analysis of distance between a given CHL1^+^ imCAF and the closest Treg cell (FOXP3^+^ CD4^+^), Tconv. cell (FOXP3^−^ CD4^+^), and CD8 T cell from (**i**) CXCR3^WT^+FOXP3^KO^ chimeric mice and (**j**) CXCR3^KO^+FOXP3^KO^ chimeric mice. White circles on graph represent mean distance of cells to imCAFs per lung tumor sampled (1 tumor per mouse sampled on 4 individual mice), grey circles on graph represent distance of an individual cell to nearest imCAF. Statistical analysis performed using one-way ANOVA on mean values (white circles). Mean and standard deviation displayed on graphs. *:0.01<p<0.05, **:0.001<p<0.01, ns; not significant. Source data

Cellular responsiveness to CXCL9, CXCL10 and CXCL11 ligand gradients is mediated by signaling through the chemokine receptor CXCR3 (ref. ^35^). While absent on naive T cells, it is well established that CXCR3 is expressed by both effector and memory T cells, as well as natural killer cells, and engagement of CXCR3 with any of its ligands leads to cell migration^36^. CXCR3-expressing T_reg_ cells have been demonstrated to play critical roles in restraining type 1 inflammation in various disease contexts, including glomerular nephritis, type 1 diabetes, multiorgan autoimmunity and subcutaneous models of cancer^37–39^. Flow cytometric profiling of T cells in lung tumors revealed that T_reg_ cells found within tumor-associated tissue exhibited the highest level of CXCR3 expression relative to other tumor T cells, as well as T_reg_ cells found within normal-adjacent and control lung tissues (Fig. 4a,b). Importantly, CXCR3^+^ T_reg_ cells were similarly detected in lung tumors arising in both KP and K_c_P genetically engineered mouse models of LUAD (Extended Data Fig. 8a–d), indicating that this T_reg_ cell subset is present across both primary and metastatic forms of lung cancer. Moreover, as compared to CXCR3^−^ tumor-associated T_reg_ cells, CXCR3^+^ tumor T_reg_ cells demonstrated increased expression of markers associated with activation (CD69), proliferation (Ki67) and immunosuppression (CTLA-4, PD-1, GITR; Fig. 4c), suggesting that CXCR3^+^ lung tumor T_reg_ cells are a more activated and immunosuppressive subset. To functionally assess the immunosuppressive capacity of CXCR3^+^ T_reg_ cells, we performed in vitro suppression assays utilizing FACS-sorted CXCR3^−^ and CXCR3^+^ T_reg_ cells (gating strategy in Supplementary Fig. 1b). CXCR3^+^ T_reg_ cells were able to suppress naive CD4^+^ T_conv_ cell proliferation to a greater extent than their CXCR3^−^ counterparts (Fig. 4d,e), suggesting that CXCR3^+^ T_reg_ cells are a distinct T_reg_ cell subpopulation with enhanced immunosuppressive capacity. Further, bulk RNA-seq of CXCR3^−^ and CXCR3^+^ tumor-associated T_reg_ cells revealed that CXCR3^+^ T_reg_ cells are transcriptionally distinct from CXCR3^−^ T_reg_ cells, as demonstrated by differential clustering by principal component analysis (Fig. 4f); moreover, CXCR3^+^ T_reg_ cells were enriched for immunosuppressive (*Il10*, *Tgfb1* and *Entpd1*) and effector (*Irf4*, *Tigit* and *Ebi3*) T_reg_ cell genes (Fig. 4g). Taken together, these findings indicate that CXCR3^+^ T_reg_ cells represent a distinct, highly immunosuppressive subset that is functionally specialized to respond to CXCL9 gradients.

### CXCR3 deficiency limits T_reg_ cell trafficking and tumor growth

**Fig. 5:**
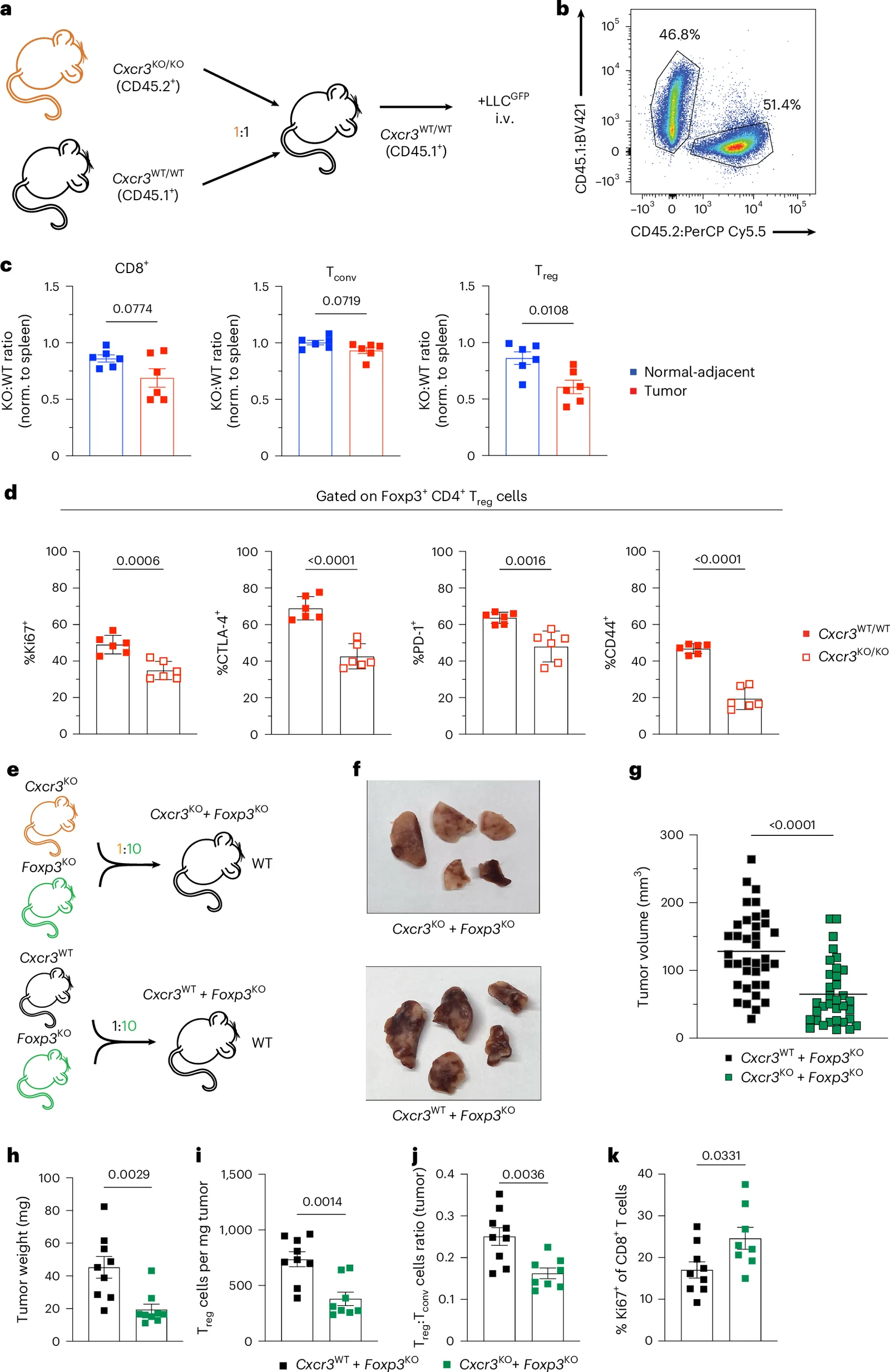
CXCR3 deficiency uniquely impairs T_reg_ cell trafficking to lung tumors and reduces tumor burden. **a**, Schematic of mixed bone marrow chimera experiment utilizing *Cxcr3*^WT/WT^ and *Cxcr3*^KO/KO^ bone marrow at a ratio of 1:1 with transfer into WT hosts. Mice were given LLC^GFP^ intravenously (i.v.) via tail vein injection 6 weeks after bone marrow transfer. **b**, Representative flow cytometry plot of congenically marked circulating immune cells found in blood of recipient mice 4 weeks after bone marrow transfer. **c**, Ratios of *Cxcr3*^KO/KO^ to *Cxcr3*^WT/WT^ for various T cell subsets (normalized to the ratio found within the spleen), normal-adjacent lung tissue and lung tumor tissue in mice with mixed bone marrow. **d**, Expression of phenotypic and functional markers on either CD45.1^+^ (*Cxcr3*^WT/WT^) or CD45.2^+^ (*Cxcr3*^KO/KO^) Foxp3^+^ T_reg_ cells. **e**, Schematic of mixed bone marrow chimera experiment utilizing *Cxcr3*^KO^ mice and *Foxp3*^KO^ mice at a ratio of 1:10 with transfer into WT C57BL/6NJ mice (males). As a control, we generated mixed bone marrow chimeras using *Cxcr3*^WT^ mice and *Foxp3*^KO^ mice at a ratio of 1:10. Mice were given LLC^GFP^ via tail vein injection 6 weeks after bone marrow transfer. **f**, Representative images of tumor-bearing lung lobes in *Cxcr3*^KO^ + *Foxp3*^KO^ transfer mice (top) and *Cxcr3*^WT ^+ *Foxp3*^KO^ transfer mice (bottom). **g**, Tumor volumes in mice of the indicated group. **h**, Cumulative weight of lung tumors in lungs of each recipient mouse. **i**, T_reg_ cells per mg of lung tumor. **j**, Ratio of T_reg_ cells to T_conv_ cells within lung tumor tissue. **k**, Frequency of Ki67^+^ cells within the CD8^+^ T cell compartment in lung tumor tissue. Statistical analysis done for flow cytometry data where two groups were compared using two-tailed unpaired *t*-tests. The mean and standard error are displayed on the graphs. **c**,**d**, Combined data from two independent experiments (*n* = 6 per group). **f**,**g**, Combined data from two independent experiments (*n* = 8 mice per group). **h**–**k**, Combined data from two independent experiments (*Cxcr3*^WT ^+ *Foxp3*^KO^ group *n* = 9, *Cxcr3*^KO ^+ *Foxp3*^KO^ *n* = 8). Source data

Given that CXCR3 plays a key role in T cell trafficking, we sought to determine whether its expression is essential for enhanced migration of all T cell subsets to the lung TME. To this end, we performed mixed bone marrow chimera experiments utilizing *Cxcr3*^KO/KO^ mice. Wild-type (WT) recipient mice received a 1:1 mixture of *Cxcr3*^KO/KO^ (CD45.2^+^) and *Cxcr3*^WT/WT^ (CD45.1^+^) bone marrow and were allowed to recover for 6 weeks before LLC^GFP^ inoculation (Fig. 5a). Evaluation of circulating lymphocytes 6 weeks after bone marrow transfer revealed an even mixture of CD45.1^+^ and CD45.2^+^ cells, suggesting that reconstitution was successful and that both *Cxcr3*^KO/KO^ and *Cxcr3*^WT/WT^ cells contributed equally to the immune compartment before tumor challenge (Fig. 5b). Strikingly, following tumor challenge, T_reg_ cells exhibited the largest reduction in the ratio of *Cxcr3*^KO/KO^ to *Cxcr3*^WT/WT^ cells among tumor-associated T cell subsets, a phenotype that was not observed for T cells isolated from normal-adjacent tissue (Fig. 5c), suggesting that loss of CXCR3 specifically impairs the migration of T_reg_ cells to the TME. Although not statistically significant, *Cxcr3*^KO/KO^ CD8^+^ T cells also demonstrated a trending level of impaired migration to the lung TME, which is consistent with a previous study using a model of subcutaneously injected colorectal cancer cells^40^. Notably, as compared to *Cxcr3*-sufficient tumor T_reg_ cells, significantly fewer tumor T_reg_ cells derived from *Cxcr3*^KO/KO^ bone marrow were positive for markers of proliferation, immunosuppression and antigen experience (Fig. 5d), suggesting that CXCR3 expression may be a critical driver of enhanced activation and immunosuppressive programs in tumor tissue-associated T_reg_ cells.

Given that CXCR3^+^ T_reg_ cells demonstrate a unique, hyper-suppressive function, we reasoned that CXCR3^+^ T_reg_ cells may be highly influential in promoting an immunosuppressive TME and poor cancer outcomes. To evaluate the effect of T_reg_ cell-specific CXCR3 expression, we performed a mixed bone marrow chimera experiment with *Cxcr3*^KO^ and *Foxp3*^KO^ bone marrow at a ratio of 1:10 (Fig. 5e). Given that *Foxp3*^KO^ bone marrow progenitors fail to produce mature T_reg_ cells^41^, all T_reg_ cells that repopulate the immune system in this context are derived from *Cxcr3*^KO^ bone marrow, while other T cell subsets are predominantly derived from *Foxp3*^KO^ bone marrow cells, and thus are WT for *Cxcr3*. As a control, we performed the same 1:10 transfer, with *Cxcr3*^WT^ and *Foxp3*^KO^ bone marrow, respectively, such that all T_reg_ cells in reconstituted animals are WT for *Cxcr3*. In this model, following LLC challenge, reconstituted mice lacking *Cxcr3*-expressing T_reg_ cells exhibited significantly lower tumor burdens relative to mice with *Cxcr3*^WT^ T_reg_ cells (Fig. 5f–h). Notably, within these tumors there was a marked reduction in the abundance of T_reg_ cells (Fig. 5i), indicating that T_reg_ cell-specific loss of *Cxcr3* greatly influences T_reg_ cell accumulation in the lung TME. Interestingly, tumors in mice with *Cxcr3*-deficient T_reg_ cells exhibited a lower T_reg_:T_conv_ ratio (Fig. 5j) and an increased proportion of proliferative CD8^+^ T cells (Fig. 5k). Supporting a functional role for CXCR3^+^ T_reg_ cells in enforcing immunosuppression, CXCR3^+^ T_reg_ cells suppressed CD8^+^ T cell proliferation more efficiently than CXCR3^−^ T_reg_ cells in an in vitro suppression assay (Extended Data Fig. 8e,f). However, CXCR3^+^ T_reg_ cells did not exhibit enhanced capacity to suppress tumor cell killing by antigen-specific CD8^+^ T cells in an in vitro cytotoxicity assay (Extended Data Fig. 8g), suggesting that, at least in vitro, CXCR3^+^ T_reg_ cells do not directly inhibit cytotoxic T lymphocyte killing. Importantly, several studies across multiple tumor models, including orthotopic lung cancer^42,43^, have demonstrated that CXCR3^+^ T_reg_ cells indirectly constrain CD8^+^ T cell cytotoxic responses through modulation of dendritic cell activation, costimulatory signaling and cytokine availability, supporting a predominantly indirect, rather than direct, mechanism of suppressing cytotoxic T lymphocyte killing by CXCR3^+^ T_reg_ cells.

Given that chemokine signaling can regulate both cellular migration and spatial positioning within tissues, we next evaluated whether T_reg_ cell-specific loss of CXCR3 altered the preferential localization of T_reg_ cells to CHL1^+^ fibroblasts. Confocal imaging and colocalization analysis of tumors from this chimeric mouse model revealed that T_reg_ cells lacking CXCR3 retained preferential spatial association with CHL1^+^ imCAFs (Extended Data Fig. 8h–j). These findings suggest that CXCR3 promotes T_reg_ cell migration to the TME, as evidenced by a lower abundance of T_reg_ cells in the TME upon CXCR3 ablation; however, the local retention or positioning of T_reg_ cells near imCAFs may be mediated through CXCR3-independent mechanisms. Together, these results identify T_reg_ cell-intrinsic CXCR3 signaling as a critical driver of T_reg_ cell accumulation and suppressive activity within the lung TME, establishing a mechanism through which CXCR3-directed recruitment can reinforce tumor-associated immunosuppression.

### Stromal Cxcl9 drives T_reg_ cell migration and tumor growth

**Fig. 6:**
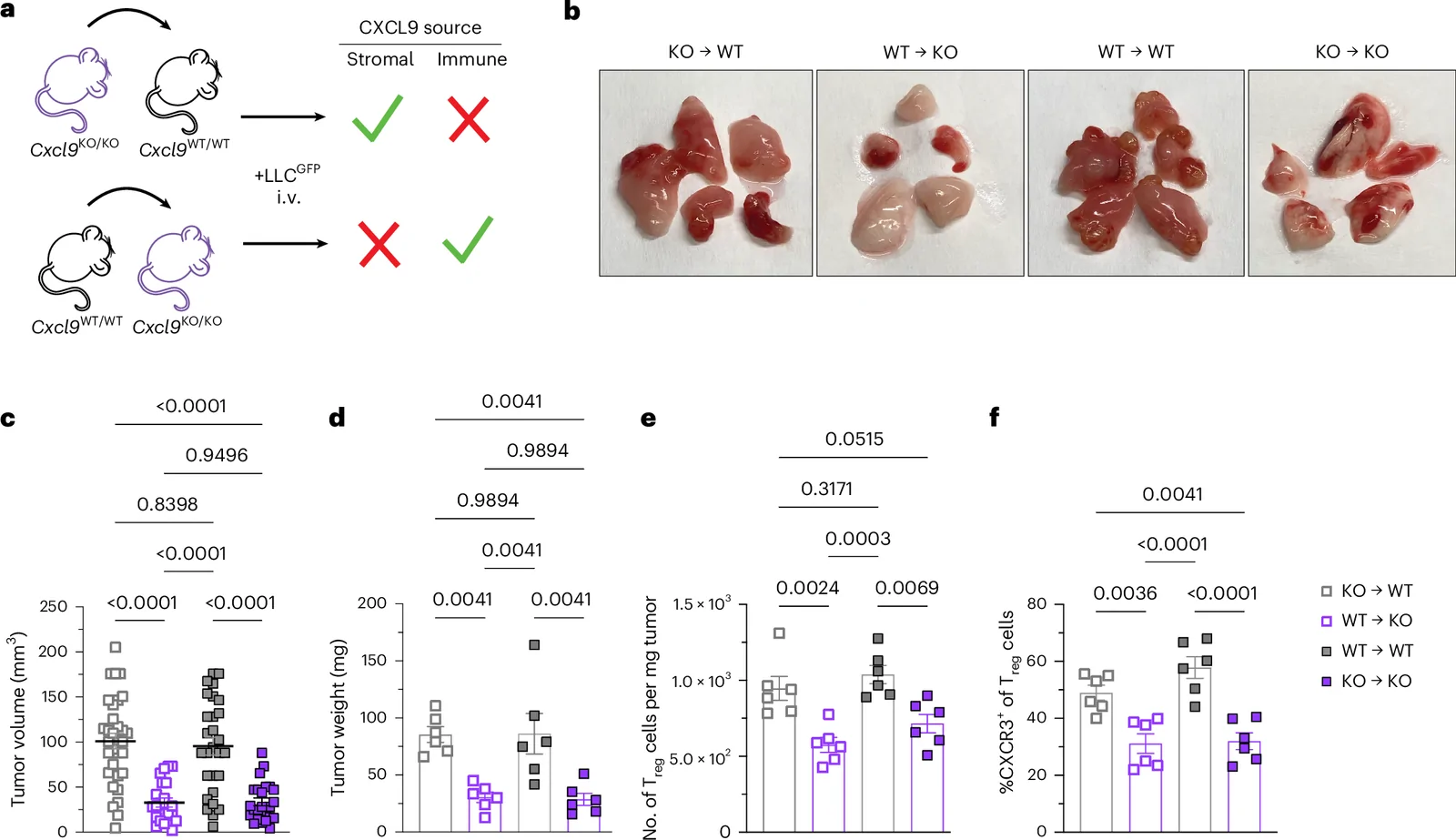
Loss of stromal-derived CXCL9 reduces hyper-suppressive T_reg_ cell migration and tumor progression. **a**, Schematic of bone marrow transfer experiment utilizing *Cxcl9*^KO/KO^ mice. As controls, *Cxcl9*^KO/KO^ bone marrow was transferred into *Cxcl9*^KO/KO^ hosts, and *Cxcl9*^WT/WT^ bone marrow was transferred into *Cxcl9*^WT/WT^ hosts (not depicted in schematic). **b**, Representative images of tumor-bearing lung lobes from indicated groups. **c**, Tumor volumes in mice of indicated group. **d**, Cumulative weight of lung tumors in lungs of each recipient mouse. **e**, Number of T_reg_ cells per mg of lung tumor tissue. **f**, Frequency of CXCR3^+^ T_reg_ cells. Statistical analysis was performed for flow cytometry data where four groups were compared using one-way ANOVA. Data were combined from two independent experiments (*n* = 6 mice per group). The mean and standard error are displayed on the graphs. Source data

**Extended Data Fig. 9:**
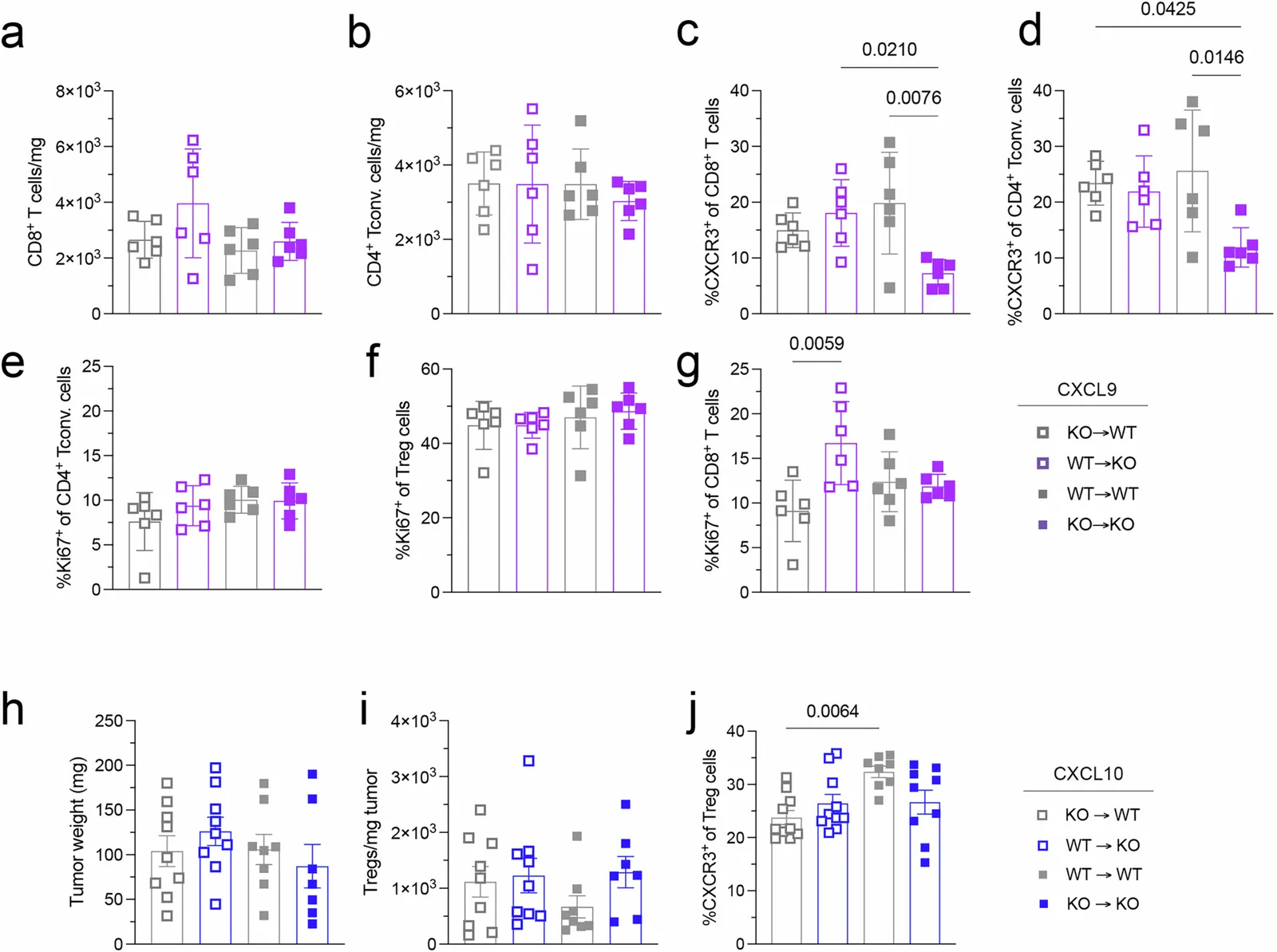
Loss of stromal-derived CXCL9 reduces CD8^+^ T cell proliferation, whereas stromal-derived CXCL10 is dispensable for Treg cell migration to the TME. **a-g**, CXCL9 bone marrow chimera experiments. Data combined from two independent experiments (n=6 mice/group). **a-b**, Number of (**a**) CD8^+^ and (**b**) CD4^+^ Tconv. cells per mg of tumor tissue. **c-d**, Frequency of CXCR3^+^ (**c**) CD8^+^ and (**d**) CD4^+^ Tconv. cells in lung tumor tissue. **e-g**, Frequency of Ki67^+^ cells in (**e**) CD8^+^ and (**f**) CD4^+^ Tconv. and (**g**) Treg cells in lung tumor tissue. (**h-j**) CXCL10 bone marrow chimera transfer experiment (KO→WT n=7, WT→KO n= 9, WT→WT n=8, KO→KO n=8). **h**, Cumulative weight of lung tumors in lungs of each recipient mouse. **i**, Number of Treg cells per mg of lung tumor tissue. **j**, Frequency of CXCR3^+^ Treg cells. Statistical analysis done for flow cytometry data comparing four groups using a one-way ANOVA. Mean and standard deviation displayed on graphs; *:0.01<p<0.05, **:0.001<p<0.01, ns; not significant Source data

Although imCAFs are potent expressors of CXCL9, hemopoietic cells such as dendritic cells and macrophages also express CXCL9 in the TME across many malignancies^32,44,45^, including lung cancer^46^, and have been implicated in dictating T_reg_ cell localization and dampening antitumor immunity^42^. However, the impact of CAF-derived CXCL9 on immune coordination and how it, in turn, may dictate tumor growth have not been explored. To evaluate if CAF-derived CXCL9 is critical for T_reg_ cell migration to the TME and if this influences cancer outcomes, we performed reciprocal bone marrow transfers between *Cxcl9*^KO/KO^ and *Cxcl9*^WT/WT^ mice (Fig. 6a). Strikingly, mice that lacked stromal expression of *Cxcl9* exhibited reduced lung tumor burden relative to mice that maintained *Cxcl9* expression in the stroma (Fig. 6b–d). Moreover, T_reg_ cell abundance was significantly reduced in tumors that lacked stromal-derived CXCL9 (Fig. 6e), whereas conventional CD4^+^ T cell and CD8^+^ T cell abundance were not significantly different across all transfer conditions (Extended Data Fig. 9a,b). Exclusive loss of hematopoietic-derived CXCL9 did not impact overall tumor burden or T_reg_ cell abundance (Fig. 6b–e), suggesting that CXCL9 produced by CAFs is critical to maintain this axis. The proportion of CXCR3^+^ T_reg_ cells found within lung tumors was reduced in all mice lacking stromal-derived CXCL9, but not within tumors that lacked hematopoietic cell-derived CXCL9 (Fig. 6f). However, proportions of CXCR3^+^ conventional CD4^+^ T cells and CD8^+^ T cells were only found to be reduced in mice that lacked both hematopoietic and stromal-derived CXCL9 (Extended Data Fig. 9c,d), suggesting that the migration of CXCR3^+^ CD4^+^ T_conv_ and CD8^+^ T cells is influenced by both hematopoietic and stromal sources of CXCL9, although this alone is not enough to drive changes in the overall abundance of these T cell subsets in the TME.

Additionally, proportions of proliferative (Ki67^+^) CD4^+^ T_conv_ cell subsets and T_reg_ cells remained unchanged across all transfer conditions (Extended Data Fig. 9e,f), whereas the proportion of proliferating CD8^+^ T cells was highest in mice lacking exclusively stromal-derived CXCL9 relative to mice that lacked exclusively hematopoietic-derived CXCL9 (Extended Data Fig. 9g). This may be indicative of greater antitumor CD8^+^ T cell expansion as a result of lower T_reg_ cell abundance in the TME, as compared with hematopoietic-only *Cxcl9* deficiency, wherein it has been demonstrated that dendritic cell-derived CXCL9 is critical for potentiating CD8^+^ antitumor immune responses^40^. To evaluate whether stromal-derived CXCL10 contributes similarly to T_reg_ cell recruitment and tumor progression, we performed reciprocal bone marrow transfer experiments using *Cxcl10*^KO/KO^ and *Cxcl10*^WT/WT^ mice (Extended Data Fig. 9h–j). In contrast to CXCL9, loss of either stromal-derived or hematopoietic-derived CXCL10 did not alter lung tumor burden or overall T_reg_ cell abundance within tumor tissue. Interestingly, the proportion of CXCR3^+^ T_reg_ cells was modestly reduced in mice lacking hematopoietic-derived CXCL10 relative to WT controls. This observation may reflect the contribution of tumor-associated myeloid cells, which in other contexts—including lymphoid tissues and sites of inflammation—produce CXCL10 to support proper T cell migration and positioning^47,48^. These findings indicate that CAF-derived CXCL9 plays a dominant and essential role in recruiting CXCR3^+^ T_reg_ cells to the TME, skewing the immune microenvironment to promote the enrichment of hyper-suppressive T_reg_ cells that prevent endogenous antitumor immunity.

### Human NSCLC imCAF-like fibroblasts predict poor survival

**Fig. 7:**
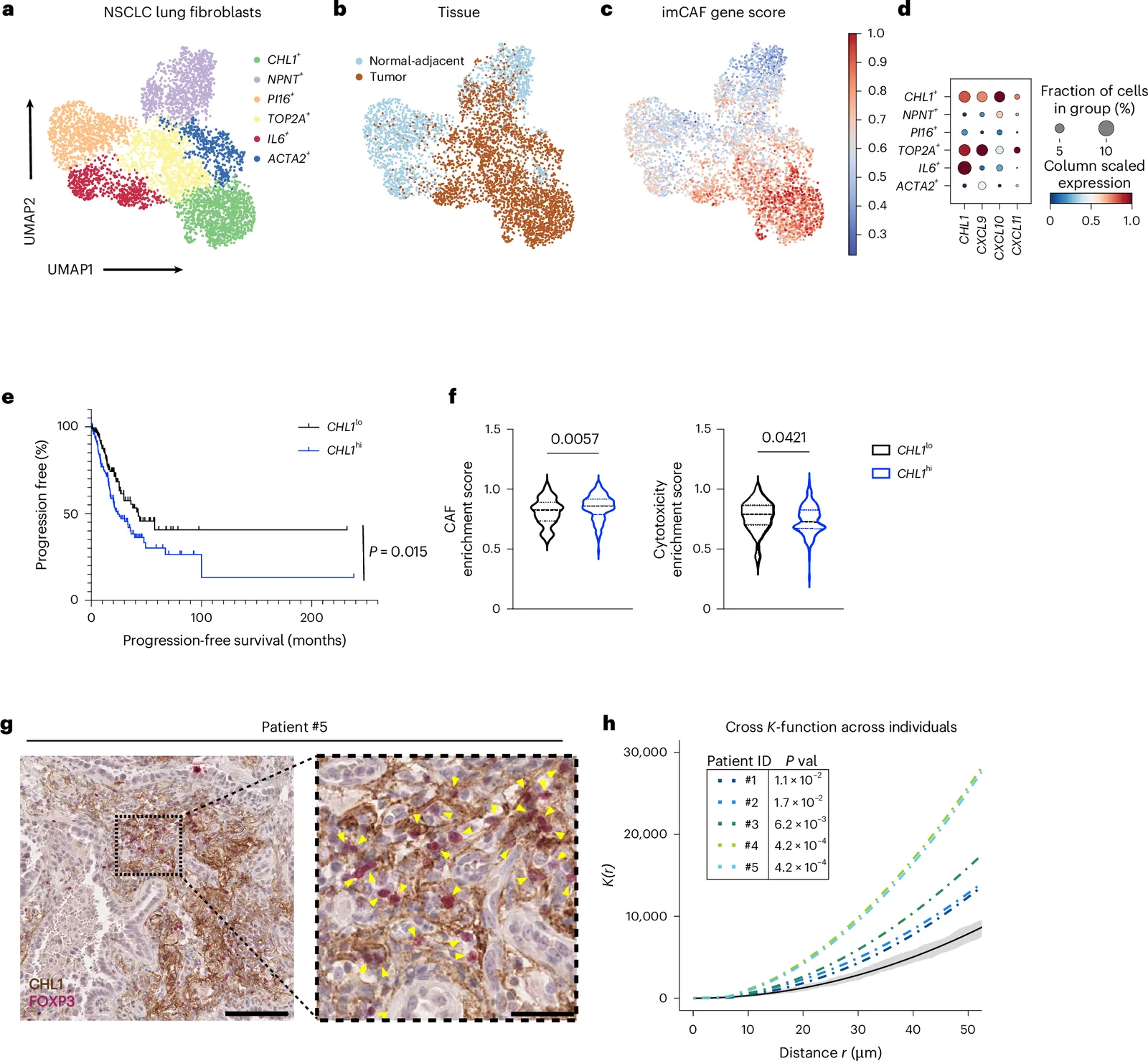
imCAF-like fibroblasts in human NSCLC are associated with T_reg_ cell localization, decreased antitumor immunity and poor survival outcomes. **a**, UMAP of 4,869 human lung fibroblasts from the single-cell lung cancer atlas comprising six clusters. **b**, UMAP highlighting the tissue of origin of fibroblasts. **c**, UMAP of mouse imCAF gene score enrichment. **d**, Dot plot of key functional and marker genes of imCAFs. **e**, Progression-free survival of individuals with high (*CHL1*^hi^) and low (*CHL1*^lo^) expression of CHL1 from the TCGA-LUAD PanCanAtlas dataset. Significance was measured using the log-rank (Mantel–Cox) test (*P* = 0.0151). **f**, Immune deconvolution utilizing ConsensusTME of *CHL1*^hi^ and *CHL1*^lo^ human gene expression data from TCGA. **g**, Representative section of lung tumor tissue from human LUAD, stained for FOXP3 (pink) and CHL1 (brown). Scale bars, 100 µm. Yellow arrowheads point to FOXP3^+^ T_reg_ cells colocalized with CHL1^+^ fibroblasts. **h**, Cross *K*-function analysis between CHL1^+^ fibroblasts and FOXP3^+^ T_reg_ cells on a human LUAD section. Statistical significance of deviations from complete spatial randomness was assessed using a Monte Carlo test with 99 simulations of a homogeneous Poisson process. Statistical analysis was performed for immune deconvolution data where two groups were compared using two-tailed unpaired *t*-tests. The mean and standard error are displayed on the graphs. Source data

**Extended Data Fig. 10:**
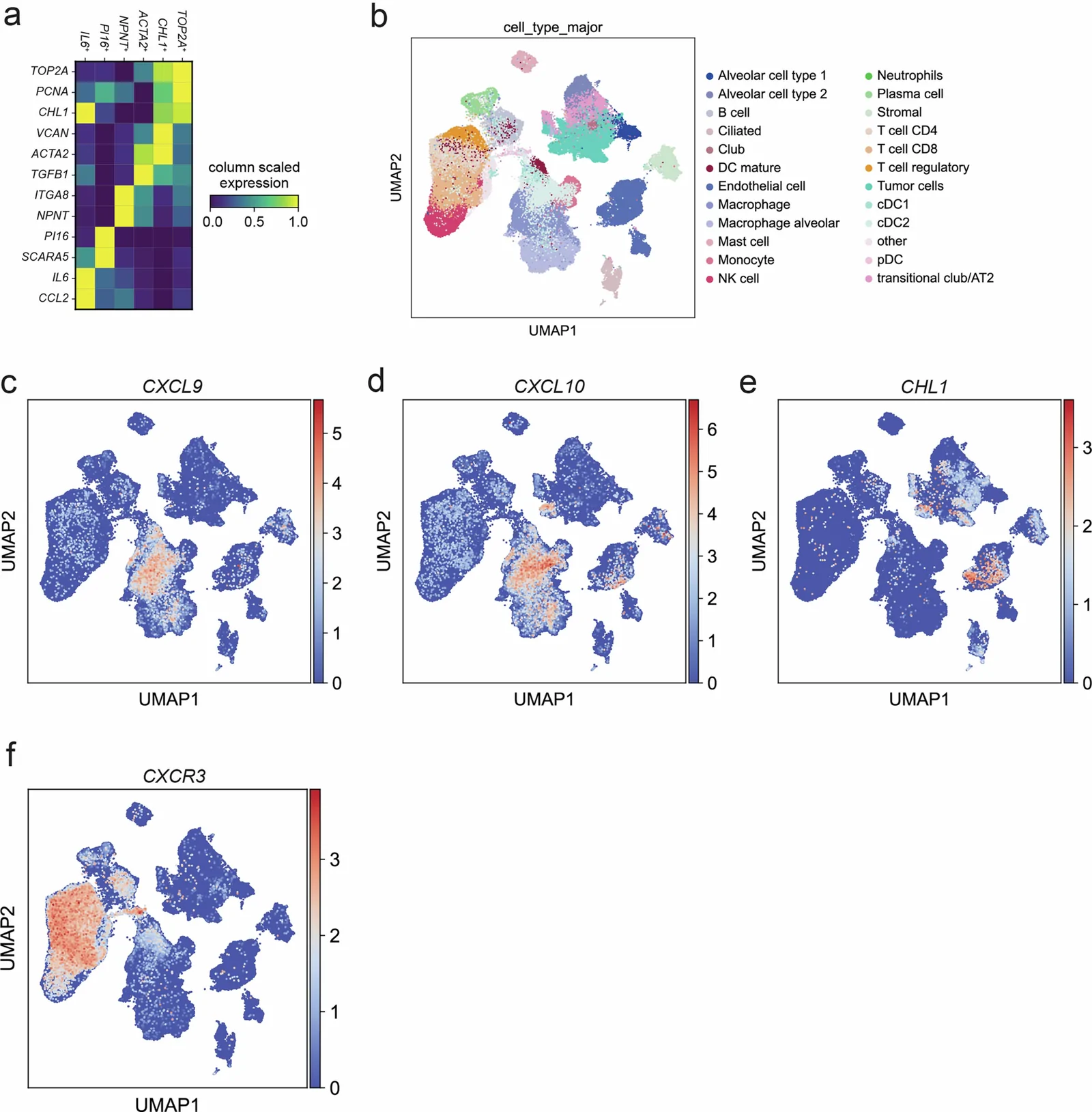
Expression of imCAF genes and CXCR3 in lung tissue of human lung cancer patients. **a**, Marker genes of fibroblast clusters. **b**, UMAP of various cell types in lung tissue of human lung cancer patients. **c**, *CXCL9*, **d**, *CXCL10*, **e**, *CHL1*, and **f**, *CXCR3* gene expression in lung tissue of human lung cancer patients.

To evaluate if CAF populations analogous to mouse imCAFs exist in human lung cancer, we utilized a large-scale non-small cell lung cancer (NSCLC) single-cell atlas^49^, sub-setting on fibroblast populations found within normal-adjacent and primary lung tumor tissue. Here, we analyzed fibroblasts from 43 different individuals across six different studies within the atlas. We resolved six transcriptionally distinct clusters, which separated by tissue type and were assigned putative identities based on hallmark gene expression patterns: *NPNT*^+^ alveolar fibroblasts^50,51^, *PI16*^+^ adventitial fibroblasts^22,51^, *IL6*^+^ CAFs^52^, *ACTA2*^+^ CAFs^1^, *CHL1*^+^ imCAFs and *TOP2A*^+^ fibroblasts expressing genes associated with proliferation (Fig. 7a,b and Extended Data Fig. 10a). Overlaying our mouse imCAF gene signature (Supplementary Table 2; top 100 DEGs) onto the human dataset revealed that the imCAF signature was highly enriched in one distinct cluster comprising tumor-associated fibroblasts (Fig. 7c). Notably, this cluster demonstrates increased expression of *CHL1*—the homologous imCAF marker identified in our mouse studies—as well as *CXCL9* and *CXCL10* (Fig. 7d), suggesting this CAF subset is phenotypically and functionally similar to mouse imCAFs. As has been previously described, gene expression analysis of all cells found within the lung TME revealed that *CXCL9* and *CXCL10* are expressed on fibroblasts, as well as conventional type 1 dendritic cells, conventional type 2 dendritic cells and macrophages (Extended Data Fig. 10b–d)^42^. *CHL1* was demonstrated to be robustly expressed on stromal cells within the TME, although some cells within the endothelial and tumor cell clusters also exhibited *CHL1* expression (Extended Data Fig. 10e). T cells were found to be the most robust expressors of *CXCR3* in the lung TME (Extended Data Fig. 10f).

Furthermore, analysis of The Cancer Genome Atlas (TCGA)-LUAD dataset revealed that individuals with high *CHL1* expression in lung tumors exhibited significantly decreased progression-free survival relative to individuals with low *CHL1* expression (Fig. 7e). Moreover, immune deconvolution of corresponding RNA-seq datasets revealed that *CHL1*^hi^ individuals exhibit a greater CAF enrichment score and decreased cytotoxicity score (Fig. 7f), suggesting that *CHL1*^hi^ individuals possess more immunologically cold, stromal cell-rich TMEs that may influence survival outcomes.

Given that we could identify analogous *CHL1*-expressing imCAFs in humans at the transcriptional level, we reasoned that we may also be able to visually identify this subset of CAFs by their expression of CHL1 at the protein level. To this end, we performed immunohistochemical staining on lung tumor resections from individuals harboring LUAD with the *KRAS* p.Gly12Cys mutation (the same mutation of the LLC^GFP^ cell line used in our mouse orthotopic model). Strikingly, co-staining for FOXP3 revealed that T_reg_ cells robustly colocalize with CHL1^+^ cells in lung tumors across five of six individuals (with one individual excluded due to >95% mucin content in the section sample; Fig. 7g,h). This spatial association strongly parallels our findings in mouse models and provides direct visual evidence that CHL1^+^ imCAF-like populations exist in human tumors and physically interact with T_reg_ cells within the lung TME. Taken together, these data not only validate the presence of imCAF-like populations in human LUAD but also highlight their potential role in orchestrating local immunosuppression through engagement with T_reg_ cells.

## Discussion

It has been well described that lung tumors harbor heterogeneous and functionally distinct CAF populations that modulate the immune microenvironment through diverse mechanisms^11,12,14,15^, yet progress has been impeded by absence of markers that cleanly define distinct subsets. The restricted expression of CHL1 on imCAFs—and its consistent association with T_reg_ cell accumulation across mouse and human tumors—establishes CHL1 as a novel biomarker for identifying individuals with LUAD who have highly immunosuppressive TMEs and who may benefit from therapies that disrupt the imCAF–T_reg_ cell axis. Moreover, our data from metastatic models indicate that imCAFs are a conserved feature of lung metastases, broadening the therapeutic relevance of this axis beyond LUAD.

CXCL9-expressing fibroblasts have been primarily characterized in secondary lymphoid organs (SLOs) as a subset of activated T-zone fibroblastic reticular cells. Stromal CXCL9 in lymph nodes spatially organizes CD4^+^ T cells to support antigen-specific immunity^31^—a function we show is subverted with striking consequences in the tumor setting. Our findings reveal that imCAF-derived CXCL9 exploits this same organizational logic to concentrate hyper-suppressive CXCR3^+^ T_reg_ cells at the tumor margin, inverting a homeostatic immune-organizing circuit into a driver of immune evasion. Notably, a subset of mouse *Cxcl9*-expressing CAFs found in pulmonary breast cancer metastases was shown to drive malignant colonization through interactions with CXCR3^+^ metastasis-initiating breast cancer cells^53^, and this population also demonstrated high *Chl1* expression—suggesting that CHL1^+^CXCL9^+^ fibroblasts constitute a conserved stromal program differentially co-opted across cancer subtypes. More broadly, the parallel between imCAFs and lymphoid-tissue stromal cells—including CCL19-producing fibroblastic reticular cells that organize CD8^+^ T cells in lung tumors^12,54^ —supports the emerging concept that tumors co-opt SLO-like stromal programs to shape adaptive immunity.

Distinct CAF–T_reg_ cell interactions have been shown to promote T_reg_ migration, activation and retention through tissue-specific mechanisms in PDAC, breast, ovarian, liver and oral cancers^5,7,17,55^, yet no defined molecular axis linking a specific CAF subset to a distinct T_reg_ population had been described in lung cancer. Our identification of the imCAF–CXCL9–CXCR3^+^ T_reg_ circuit fills this gap, revealing a stromal mechanism of immune evasion in LUAD and a potentially targetable node for immunotherapy. Relatedly, efforts to specifically target T_reg_ cell subsets associated with enhanced trafficking in solid cancers, such as CCR4^+^ or CCR8^+^ T_reg_ cells, are being explored clinically^56,57^. Given the role of T_reg_ cells in pulmonary tolerance to airborne antigens, selective targeting of hyper-suppressive CXCR3^+^ T_reg_ subsets—rather than global depletion—may improve antitumor immunity while limiting systemic toxicity.

Recent work has shown that CXCR3^+^ T_reg_ cells uniquely localize to CXCL9-expressing dendritic cells in subcutaneous colorectal cancer models, suppressing cross-presentation and antitumor immunity^42^. Our orthotopic findings extend this model: CAF-derived CXCL9—not only dendritic cell-derived—is a critical source driving CXCR3^+^ T_reg_ recruitment, a contribution that subcutaneous models are poorly positioned to capture. Thus, analogous, CXCL9-expressing imCAF populations may exist across solid tumor types and coordinate CXCR3^+^ T_reg_ recruitment more broadly.

CAFs have been proposed to organize immune cells through multi-step processes involving chemokine-directed trafficking and niche-specific retention^5^. Our data support this framework in the lung: CXCL9–CXCR3 signaling drives T_reg_ migration into the TME, yet CXCR3 loss does not disrupt preferential T_reg_ positioning near CHL1^+^ imCAFs, implying that separate imCAF-derived cues mediate local retention. Future work will be required to define these retention signals and determine whether disrupting them can further enhance antitumor immunity.

CXCR3 expression in T_reg_ cells has been shown to be directly regulated by the transcription factor T-bet. Therefore, many studies that evaluate functional differences of CXCR3^+^ T_reg_ cells do so through T_reg_ cell-specific deletion of T-bet^37–39^, demonstrating that CXCR3^+^ T_reg_ cells restrain autoimmune pathology. These earlier findings raise the question of whether stromal CXCL9 coordinates hyper-suppressive T_reg_ trafficking in autoimmune contexts as it does in tumors.

Our findings reframe how we think about stromal control of intratumoral immunity: rather than acting merely as structural scaffolding, fibroblasts can function as active organizers of immunosuppressive T cell circuits—co-opting lymphoid-tissue-like programs to concentrate hyper-suppressive T_reg_ cells at the tumor margin. This stromal–immune axis provides a rationale for therapeutic strategies that selectively disrupt imCAF–CXCL9–CXCR3^+^ T_reg_ cell recruitment, potentially enhancing checkpoint blockade efficacy in LUAD and other desmoplastic malignancies without the systemic toxicity of global T_reg_ cell depletion.

## Methods

### Mice

Animal experiments were conducted with the approval of Columbia University’s Institutional Animal Care and Use Committee (protocol AC-AABT2656). The mice were housed in a facility under a 12-h light–dark cycle, with an ambient temperature of 20–25 °C and humidity maintained between 30% and 70%. For all experiments, mice were on the C57BL/6 background and examined at 8–12 weeks old. C57BL/6NJ (JAX stock no. 005304), *Cxcr3*^KO^ (JAX stock no. 005796), *Cxcl10*^KO/KO^ (JAX stock no. 006087), *OT1* (JAX stock no. 003831) and B6 CD45.1 (JAX stock no. 002014) were obtained from Jackson Laboratories. *Cxcl9*^KO/KO^ mice were a generous gift from the laboratory of A. D. Luster (Massachusetts General Hospital; C57BL/6J background). *Foxp3*^EGFP^ and *Foxp3*^ΔEGFPiCre^ (*Foxp3*^KO^) male mice on the C57BL/6J background were a generous gift from the laboratory of A. Rudensky (Memorial Sloan Kettering Cancer Center). *Kras*^LSL-G12D/+^*; Trp53*^fl/fl^*; Rosa26*^LSL-TdTom/+^ (‘KP mice’) and *Kras*^LSL-G12C/+^; *Trp53*^fl/fl^*; Rosa26*^LSL-TdTom/+^ (‘K_c_P mice’) were a generous gift from the laboratory of C.P.C.-C. (Columbia University Irving Medical Center). All experiments were performed with female mice unless otherwise noted.

### Cell lines and metastatic modeling

LLC cells were obtained from the American Type Culture Collection (CRL-1642). GFP was transfected to into LLC cells using cDNA obtained from AddGene (60206). KPCy, B16F10 and B16F10^GFP^ cell lines were a generous gift from the lab of R. Schwabe (Columbia University Irving Medical Center). All cell lines were grown in DMEM supplemented with 10% FBS, 1% L-glutamine, 1% penicillin–streptomycin and 10 mM HEPES (pH 7.2–7.5). Cultures were maintained in a humidified 37 °C, 5% CO_2_ incubator. For metastatic colonization of cancer cells, cells were harvested in 0.05% trypsin, washed, counted and resuspended in DMEM without serum at a concentration of 7.5 × 10^5^ cells per ml. Unless otherwise stated, female mice were injected with 1.5 × 10^5^ cells at a volume of 200 µl via tail vein injection. Lung tumors and normal-adjacent tissue were then assessed 17 days after intravenous injection. Animals were monitored daily according to Institutional Animal Care and Use Committee-approved humane endpoint criteria, including respiratory distress, marked lethargy, loss of the righting reflex, >20% body weight loss, severe dehydration or inability to ambulate. No animals exceeded these approved humane endpoint criteria.

### Immunofluorescence microscopy

Mice harboring tumor-bearing lung lobes were perfused with 10 ml of 4% paraformaldehyde solution through the heart. Lung lobes were then fixed in 5 ml of 4% paraformaldehyde solution in PBS at 20–25 °C and away from light. After fixation, lung lobes were placed in a 30% sucrose solution and allowed to dehydrate for 48 h at 4 °C. Lobes were then snap frozen and embedded in optimal cutting temperature compound (Fisher Healthcare, 23-730-571). Tissue sections were then cut onto charged (Fisher Scientific, 12-550-15), poly-D-lysine (Sigma, P4707)-coated slides at a thickness of 8 µm and fixed in ice-cold acetone solution for 10 min. Antigen retrieval was performed (Vector Laboratories, H-3301-250) and slides were blocked with horse serum (Gibco, 16050122) or goat serum (Jackson ImmunoResearch, 005000121) in PBS + 0.3% Triton X-100 for 1h at 20–25 °C. For primary antibody staining, we utilized the following antibodies: rabbit anti-CD4 (Abcam, ab183685) and rat anti-Foxp3 (Invitrogen, 14577382), rabbit anti-CD8 (Abcam, ab217344), rabbit anti-GFP (Invitrogen, A21311), Syrian hamster anti-podoplanin (AngioBio, 11-033), goat anti-CHL1 (R&D, AF2147), rat anti-CHL1 (R&D, MAB2147), rat anti-CXCL9 (R&D, AF-492), rat anti-Tenasin C (R&D, MAB2138) and mouse anti-smooth muscle actin (Sigma, C6198). All primary antibody staining was done in PBS + 0.3% Triton X-100 with 2% horse or goat serum at 4 °C overnight in the dark. Secondary staining was performed using donkey anti-rat AF488, donkey anti-rabbit Cy5, donkey anti-goat Cy5, donkey anti-goat Cy3, donkey anti-rat Cy3 and goat anti-Syrian hamster Cy5 (Jackson ImmunoResearch). Slides were then imaged on a Nikon AXR MP Confocal microscope at ×20 magnification. Quantification of the distance between Foxp3^+^ CD4^+^, Foxp3^−^ CD4^+^ and CD8^+^ T cells to CHL1^+^, CHL1^+^CXCL9^+^ or CHL1^−^CXCL9^+^ cells was performed using a nearest-neighbor search algorithm in R.

### Human lung immunohistochemistry staining

Tissue was retrieved from the Columbia University Irving Medical Center Database Shared Resources/Columbia University Tumor Bank (IRB no. AAAB267). Formalin-fixed, paraffin embedded lung resection samples were obtained from LUADs with confirmed *KRAS* p.Gly12Cys mutations in compliance with protocols approved by our Institutional Review Board. All samples received were included in our analysis except for one, which comprised predominantly (>95%) mucin and scant neoplastic epithelium. Lung tumor tissue was then serially sectioned at a thickness of 8 μm onto positively charged glass slides and stored at 4 °C. Slides were stained using immunohistochemistry methods by the Columbia Molecular Pathology Core for CHL1 (Abcam, ab106269) and FOXP3 (Invitrogen; clone 236A/E7; 14477782). Tile scans of slides were performed at ×20 on a Leica AT2 scanning microscope. Visualization of slides was done on QuPath 0.5.1 and Fiji (version 1) software programs.

### Localization analysis of T_reg_ cells and imCAFs in humans

We used QuPath software standard tools to perform thresholding and identify the spatial coordinates of CHL1^+^ CAFs and FOXP3^+^ T_reg_ cells. Cell centroid coordinates were extracted from segmented imaging data and treated as spatial point patterns within the defined tissue region of interest. To evaluate deviations from spatial randomness, the observed *K*-function was compared to simulations generated under a complete spatial randomness null model, implemented as a homogeneous Poisson process. For each analysis, 99 Monte Carlo simulations were generated within the same spatial window and with the same number of points as observed in the empirical dataset. Statistical significance was assessed using a Monte Carlo hypothesis test. To account for boundary effects inherent to spatial point pattern analysis, edge corrections were applied during *K-*function estimation. Analyses were performed using functions implemented in the spatstat package in R^58^.

### Flow cytometry

For flow cytometric analysis of cells, viability of cells was checked using either Ghost Dye viability reagent (Cytek) or Zombie Violet Fixable Viability Dye (BioLegend). Cells were then incubated with FC block (purified anti-mouse CD16/CD32, clone 2.4G2; Cytek) in flow cytometry buffer (1× PBS with 1% BSA, 2.5 mM EDTA and 0.1% sodium azide) for 10 min on ice before all surface staining with directly conjugated antibodies listed in Supplementary Table 4. For intracellular staining, cells were fixed/permeabilized with Foxp3/Transcription Factor Staining Buffer Kit (Cytek, TNB-0607-KIT) and stained with directly conjugated antibodies (Supplementary Table 4). Surface staining of CHL1 was performed before using directly conjugated antibodies.

For surface staining of CHL1, cell suspensions were blocked in 10% horse serum (Gibco, 16050122), washed and incubated with rat anti-CHL1 (1:200 dilution; clone MAB2126; R&D) in 2% horse serum at 4 °C for 30 min. Cells were then washed twice and stained with mouse anti-rat AF647 secondary antibody (Invitrogen, A48272) in 2% horse serum for 15 min on ice. Fluorescence was examined with a BD LSRFortessa and analyzed on FlowJo v.10.8.1

### Chemotaxis assay

CD90.2^+^ cells were isolated from the spleen, lung tumor tissue and normal-adjacent lung tissue cells by positive bead selection (Supplementary Methods). Cells were then counted, normalized across samples and placed in the top portion of a 5-µm Transwell insert in a 96-well TC plate (Corning, 3388) or in a well with no Transwell insert. In total, 235 µl of RPMI 1640 supplemented with 5% FBS, 1% L-glutamine, 1% penicillin–streptomycin, 1% NEAA, 1% sodium pyruvate, 55 µM BME and 10 mM HEPES (pH 7.2–7.5), with or without 100 ng ml^−1^ recombinant mouse CXCL9 (Fisher Scientific, 492-MM-010) or CXCL10 (Fisher Scientific, 466-CR) was placed on the bottom portion of the Transwell. Cells were incubated at 37 °C for 3 h. Cells were then collected from the bottom chamber of the Transwell and analyzed by flow cytometry. Cell migration was calculated using the following formula: Cell migration = (number of no insert events − number of events in condition) / number of no insert events.

### CXCR3^−^ versus CXCR3^+^ T_reg_ cell suppression assay

T_reg_ cells were isolated from the spleen and lymph nodes of male Foxp3^GFP^ mice using CD4^+^ T cell negative enrichment protocols (Supplementary Methods). Foxp3^+^CXCR3^+^, Foxp3^+^CXCR3^−^ and CD4^+^ CD62L^+^CD44^−^ naive T_conv_ or CD8^+^ T cells were sorted using the BD FACSAria II (Supplementary Methods). Additionally, splenocytes from a separate Foxp3^GFP^ mouse were irradiated (20 Gy) and supplemented with anti-CD3 activating antibody at 2× concentration (2 µg ml^−1^) to serve as antigen-presenting cells. T_conv_ cells were labeled with 2 µM of CFSE labeling dye (Cytek, 13-0850-U500). Either Foxp3^+^CXCR3^+^ or Foxp3^+^CXCR3^−^ T_reg_ cells were plated at 4 × 10^4^ cells per well in a 96-well plate in triplicate. T_reg_ cells were then serially diluted to achieve a range of T_reg_ cell concentrations (4 × 10^4^–0 cells per well). For evaluating the suppressive capacity of T_reg_ cell populations on T_conv_ cells, T_conv_ cells and irradiated splenocytes were plated at a final concentration of 1 × 10^5^ cells per well and 4 × 10^4^ cells per well, respectively, with T_reg_ cells and cocultured for 72 h in RPMI 1640 supplemented with 5% FBS, 1% L-glutamine, 1% penicillin–streptomycin, 1% NEAA, 1% sodium pyruvate, 55 µM BME and 10 mM HEPES (pH 7.2–7.5). For evaluating the suppressive capacity of T_reg_ cell populations on CD8^+^ T cells, CD8^+^ T cells were plated at 4 × 10^4^, and T_reg_ cell numbers were adjusted to match the appropriate ratio for a given condition. Irradiated splenocytes were plated at a final concentration of 1 × 10^5^. Cells were then analyzed by flow cytometry to evaluate T_conv_ cell or CD8^+^ T cell proliferation by CFSE labeling.

### scRNA-seq

For the fibroblast-enriched dataset, mouse LLC^GFP^-bearing lungs (*n* = 3 mice) were dissected, microdissected to separate tumor and normal-adjacent lung tissue, labeled with hashtags based on tissue designation and mouse number (TotalSeq-B0301–TotalSeq-B0306; BioLegend; 1:200 dilution; one hashtag per sample, *n* = 6 samples total; 3 lung tumor tissue and 3 normal-adjacent lung tissue), and sorted as described (Supplementary Methods). For the full tumor-bearing lung homogenate reference dataset, LLC^GFP^-bearing lungs (*n* = 3) were dissected and marked with the aforementioned hashtags (TotalSeq-B0301–TotalSeq-B0306; BioLegend; 1:200 dilution). At the Columbia Genome Center, libraries were prepared using the 10x Genomics Single Cell Gene Expression 3′ workflow, and sequencing was performed on an Illumina NovaSeq 6000, followed by processing using Cell Ranger 6.1.2. Analysis was then performed using Python 3.9 and Scanpy^59^ version 1.10 standard commands. To compute PCA coordinates and variance ratio, both n_comps and n_pcs were set to 80. To compute the nearest-neighbors distance matrix and a neighborhood graph of observations, n_neighbors was set to 15, and n_pcs was set to 40. The Leiden algorithm was used to perform clustering, with resolution set to 0.4. REACTOME pathway analysis was performed utilizing the DecoupleR 1.4.0 package (Python version).

### Spatial transcriptomics

Mouse lung lobes bearing LLC^GFP^ tumors (*n* = 2) were embedded in optimal cutting temperature compound as fresh frozen tissue and sectioned onto 10x Visium Spatial Gene Expression slides at 8-µm thickness. Sections were then permeabilized and processed to obtain cDNA libraries that were quality controlled using an Agilent Bioanalyzer. The cDNA libraries were sequenced on the Illumina NovaSeq X. Spatial transcriptomic slides were analyzed using Python 3.9. Reference-based spatial deconvolution with mappable, concatenated scRNA-seq (enriched LLC^GFP^ tumor-bearing lung fibroblast scRNA-seq dataset and whole lung tumor homogenate scRNA-seq dataset) was performed using the cell2location pipeline with standard commands^60^. In this framework, cell-type-specific reference signatures are derived from the average gene expression profiles of annotated scRNA-seq clusters. A hierarchical Bayesian model is then used to decompose the spatial transcriptomic counts at each Visium capture location into probabilistic mixtures of these reference signatures, thereby estimating the abundance and spatial distribution of each cell population. Inference of T cell-subset localization was performed utilizing the reference-free deconvolution method Starfysh (Supplementary Table 1)^61^. The epoch number was set to 500 to ensure training convergence, with other parameters reported as default. SCI values^62^ were computed to quantify spatial cross-correlation between the inferred cell-type compositions. The implementation of SCI was modified upon Starfysh with Python modules Scanpy^59^ version 1.10 and Squidpy^63^ version 1.6.2, where a neighborhood graph was constructed between each spot and its three-hop nearest neighbors along the hexagonal coordinate.

### T_reg_ cell RNA-seq and analysis

Foxp3^GFP^ male mice were i.v. injected with LLC cells and lung tumor tissue was dissected (Supplementary Methods). Tissues were then negatively enriched for CD4^+^ T cells, and both CXCR3^−^ and CXCR3^+^ T_reg_ cells were FACs sorted (Supplementary Methods) separately into TRIzol LS (Fisher, 10-296-010). RNA was extracted by the Columbia Molecular Pathology Core using an Agilent RNA 6000 Pico Kit, with RIN scores > 9.8. RNA-seq of T_reg_ cells was performed by the Columbia Genome Center utilizing poly-A pulldown techniques to isolate RNA, with subsequent library preparation with a Nextera XT Kit (Illumina). Libraries were then sequenced using the Element AVITI system at 40 million reads. Pseudoalignment was performed using a kallisto index (version 0.44.0) from transcriptomic data (Ensembl v96, Mouse GRCm38.p6). Differential gene expression analysis of RNA-seq was done with DESeq-2 in R using standard commands.

### Human scRNA-seq analysis

We used the publicly available single-cell lung cancer atlas dataset. Cells were extracted for analysis based on fibroblast cell-type designation (cell_type = ‘fibroblasts’) that were sequenced using Illumina 10× 3′ V3 sequencing and derived from normal-adjacent or tumor tissue from individuals with LUAD, squamous cell lung carcinoma and NSCLC. We then performed standard integration and clustering using Harmony and Scanpy workflows and standard commands. To compute PCA coordinates and variance ratio, both n_comps and n_pcs were set to 50. To compute the nearest-neighbors distance matrix, we used Harmony integration, with max_iter_harmony set to 500. To compute neighbors, use_rep was set to ‘X_pca_harmony’. The top 100 DEGs from our mouse imCAF cluster were converted to human gene IDs and were used to compute a gene score for expression of the gene set within the human fibroblast dataset (Supplementary Table 2).

### TCGA survival analysis and immune deconvolution

We used the cBioPortal (https://www.cbioportal.org/) to access the TCGA-LUAD PanCanAtlas dataset. We extracted gene expression data and metadata from individuals within the top and bottom 25% of *CHL1* gene expression range within the dataset. The results herein are based upon data generated by the TCGA Research Network (https://www.cancer.gov/tcga/).

We used the Immunedeconv^64^ package in R to achieve immune deconvolution and scoring of individuals in the *CHL1*^lo^ and *CHL1*^hi^ categories with standard commands. Immune deconvolution scores were computed using ConsensusTME^65^ enrichment scoring methodology.

### Statistics and reproducibility

No statistical methods were used to predetermine sample sizes. Sample sizes were selected based on previous experience with these experimental models and are comparable to those reported in previous publications^5,20^. No data were excluded from the analyses. Experiments were not randomized. Data collection and analysis were not performed blind to the conditions of the experiments. Data distribution was assumed to be normal, but this was not formally tested. Statistical analyses and data visualization were performed using GraphPad Prism (v10.1.2), R, RStudio and Python (v3.9), unless otherwise stated. Biological replicates represent independent samples derived from distinct animals or independent cell preparations, as indicated in the figure legends.

### Inclusion and ethics statement

Before surgery, all patients had given surgical informed consent, which was approved by the Columbia University Institutional Review Board (no. AAAB2667). Lung tumor tissue was retrieved from the Columbia University Irving Medical Center Database Shared Resources/Columbia University Tumor Bank. Samples were de-identified before receipt in accordance with our Institutional Review Board protocol (no. AAAB2667).

### Reporting summary

Further information on research design is available in the Nature Portfolio Reporting Summary linked to this article.

## Online content

Any methods, additional references, Nature Portfolio reporting summaries, source data, extended data, supplementary information, acknowledgements, peer review information; details of author contributions and competing interests; and statements of data and code availability are available at https://doi.org/10.1038/s41590-026-02607-2.

## Supplementary information

Supplementary Information Supplementary Figs. 1 and 2, and Methods.

Reporting Summary

Supplementary Tables 1–5

## Source data

Source Data Fig. 2 Statistical source data.

Source Data Fig. 3 Statistical source data.

Source Data Fig. 4 Statistical source data.

Source Data Fig. 5 Statistical source data.

Source Data Fig. 6 Statistical source data.

Source Data Fig. 7 Statistical source data.

Source Data Extended Data Fig. 1 Statistical source data.

Source Data Extended Data Fig. 2 Statistical source data.

Source Data Extended Data Fig. 5 Statistical source data.

Source Data Extended Data Fig. 6 Statistical source data.

Source Data Extended Data Fig. 7 Statistical source data.

Source Data Extended Data Fig. 8 Statistical source data.

Source Data Extended Data Fig. 9 Statistical source data.

## Data Availability

RNA-seq, scRNA-seq and spatial transcriptomics data generated in this study have been deposited in NCBI’s Gene Expression Omnibus under accession number GSE303166. Source data are provided with this paper.

## Code availability

We report no original source code from this paper.
