# Author Correction: A novel CAF population coordinates hyper-suppressive regulatory T cell recruitment and localization in lung cancer

**DOI:** 10.1038/s41590-026-02680-7

**Published:** 2026-09-10

**Authors:** Olivia R. Ringham, Monica Rivera, Lucas F. Loffredo, Melih Arda Ozsoy, Christina M. Healy, Maye F. Cheng, Yinuo Jin, Noah Chen, Kenia de los Santos-Alexis, Elham Azizi, Anjali Saqi, Matthew B. Buechler, Carla P. Concepcion-Crisol, Nicholas Arpaia

**Affiliations:** 1 Department of Microbiology & Immunology, Columbia University Irving Medical Center, New York, NY USA; 2 Herbert Irving Comprehensive Cancer Center, Columbia University Irving Medical Center, New York, NY USA; 3 Department of Immunology, University of Toronto, Toronto, Ontario Canada; 4 Department of Biomedical Engineering, Columbia University, New York, NY USA; 5 Irving Institute for Cancer Dynamics, Columbia University, New York, NY USA; 6 Department of Pathology and Cell Biology, Columbia University Irving Medical Center, New York, NY USA

**Keywords:** Tumour immunology, Translational immunology

Correction to: *Nature Immunology* https://doi.org/10.1038/s41590-026-02607-2, published online 11 August 2026.

In the version of the article initially published, in the second paragraph of the “CXCR3 deficiency limits T_reg_ cell trafficking and tumor growth” section, the text “Interestingly, tumors in mice with *Cxcr3*-sufficient T_reg_ cells exhibited a higher T_conv_:T_reg_ ratio” should have read “Interestingly, tumors in mice with *Cxcr3*-deficient T_reg_ cells exhibited a lower T_reg_:T_conv_ ratio”. This correction has been made to the HTML and PDF versions of the article.

