# Supplementary material for: A novel CAF population coordinates hyper-suppressive regulatory T cell recruitment and localization in lung cancer
Source: Nat Immunol. 2026 Aug 11;27(9):1899–912. doi: 10.1038/s41590-026-02607-2 (PMC13506330; doi:10.1038/s41590-026-02607-2)
Supplement: Supplementary file 1 [file 41590_2026_2607_MOESM1_ESM.pdf]

nature immunology

Supplementary information https://doi.org/10.1038/s41590-026-02607-2

A novel CAF population coordinates hyper-suppressive regulatory T cell recruitment and localization in lung cancer

In the format provided by the authors and unedited

Supplementary Figure 1

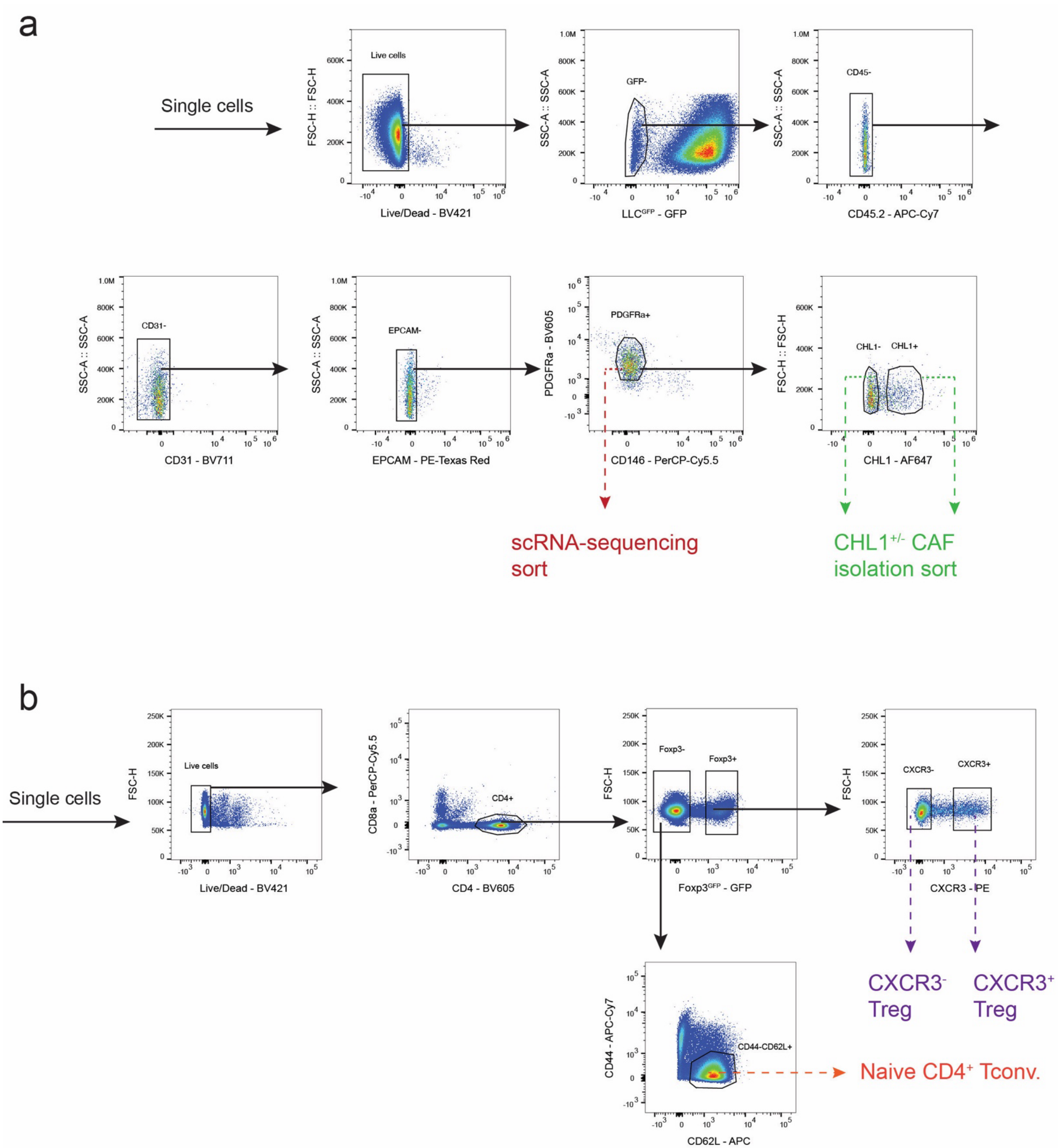

Supplementary Figure 1: Gating strategies for sorting fibroblast and Treg cell/Tconv. cell subsets

a, Gating strategy for isolation of fibroblasts from tumor bearing lung lobes for scRNA-sequencing (red) and isolation of CHL1- and CHL1+ CAFs from tumor tissue (green) for gene expression validation by RT-qPCR. **b,** Gating strategy for isolation of CXCR3- Treg cells, CXCR3+ Treg cells (purple), and naïve CD4+ Tconv. cells (orange) for Treg suppression assays and bulk RNA sequencing of CXCR3- and CXCR3+ Treg cells from Foxp3GFP male mice.

Supplementary Figure 2

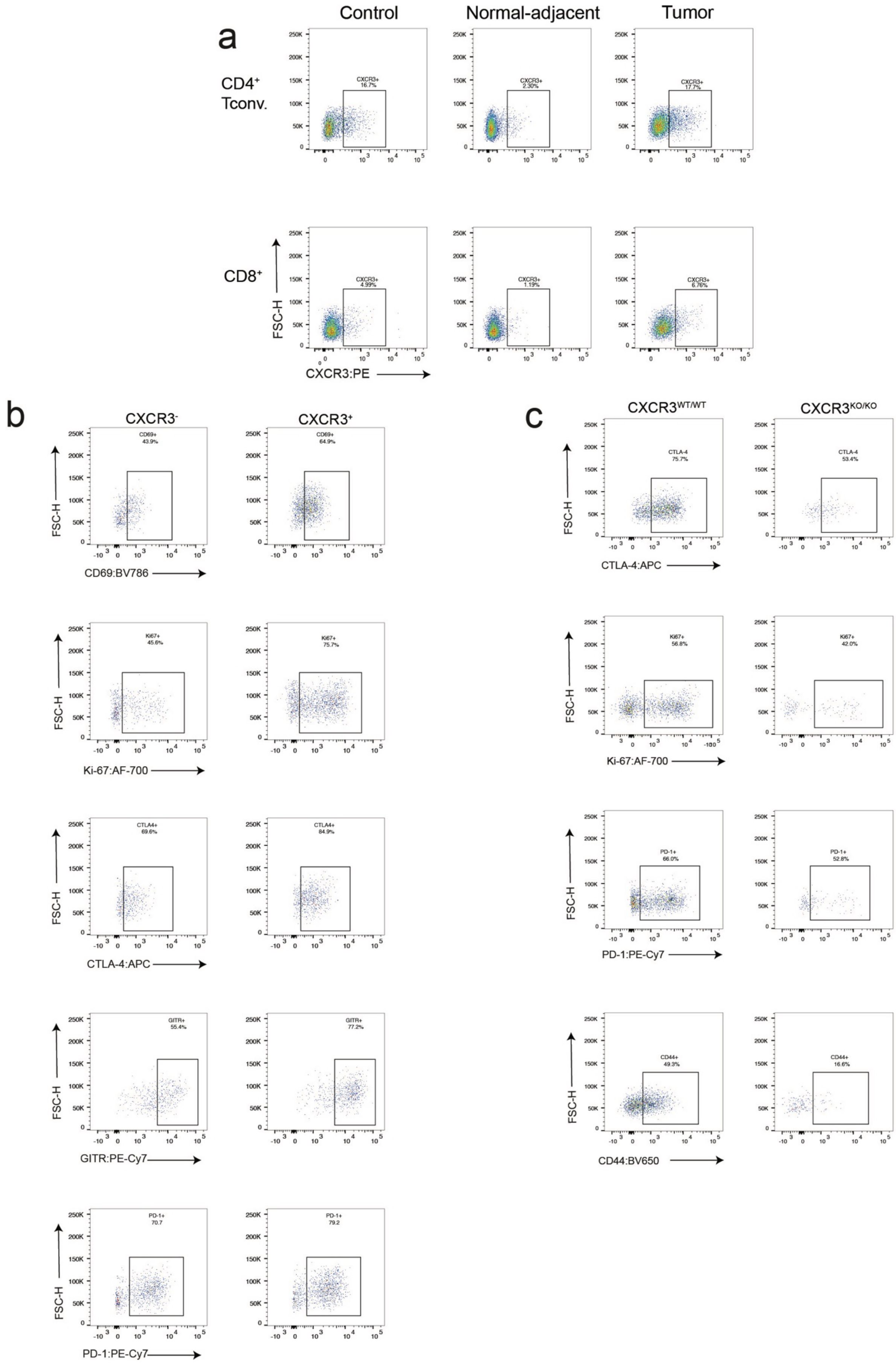

Supplementary Figure 2: Representative flow cytometry plots

a, Representative flow cytometry plots for quantified experiments associated with Fig. 4b, **(b)** Fig. 4c, and **(c)** Fig. 5d.

Supplementary Methods

Lung tumor/normal adjacent tissue processing

Mice were euthanized utilizing CO_2 euthanasia and dissected to expose the lungs and heart. Tumor-bearing lung lobes from mice were perfused with cold PBS through the heart. Tumor and normal adjacent lung tissue was separated utilizing microdissection techniques. Tissues were then finely chopped and placed in tubes containing disassociation media (RPMI with 1% l-glutamine, 1% penicillin-streptomycin, 10 mM HEPES (pH 7.2-7.5) [all Gibco], and 5% FBS [Corning]) and digestion enzymes (5 U/mL DNAse, 1 mg/mL of collagenase A, and 1 mg/mL of dispase). Tissues were then digested at 150 rpm in a 37°C agitation chamber for 45 min. Digested tissues were then ground over a 100 µm mesh cell strainer and rinsed with 15 mL tissue preparation media. Cells were then pelleted (450 x g/4°C/5 min), lysed with ACK lysis buffer (deionized water with 154 mM ammonium chloride, 10 mM potassium bicarbonate, 0.1 mM ethylenediaminetetraacetic acid [EDTA] disodium salt dihydrate, pH 7.2), and strained through 100 µm nylon mesh. Cells were pelleted again and resuspended in appropriate concentrations for antibody staining for FACS or magnetic enrichment protocols.

$CD90.2^+$ T cell positive bead enrichment

Digested spleen, tumor and normal-adjacent lung tissue cell suspensions washed twice in PBS. $CD90.2^+$ positive magnetic bead enrichment was performed according to manufacturer instructions (Invitrogen Cat# 11465D).

CAF or $CD4^+$ T cell negative bead enrichment

Digested lung tumor tissue cell suspensions were washed and incubated with FC block (1:200; purified anti-mouse CD16/CD32, clone 2.4G2; Cytek), followed by incubation with an antibody cocktail comprised of biotinylated antibodies at 2X concentration. For negative enrichment of CAFs or normal-adjacent fibroblasts, we utilized antibodies towards mouse CD45 (1:200; clone 30-F11; BioLegend), CD31 (1:200; clone 390; BioLegend), Epcam (1:200; clone G8.8; BioLegend), and TER-119 (1:200;

clone TER-119; BioLegend). For negative enrichment of CD4$^+$ T cells, we utilized biotinylated antibodies towards mouse CD31 (1:200; clone 390; BioLegend), Epcam (1:200; clone G8.8; BioLegend), TER-119 (1:200; clone TER-119; BioLegend), Pdgfrα (1:200; clone APA5; BioLegend), CD19 (1:200; clone 6D5; BioLegend), NK1.1 (1:200; clone PK136; BioLegend), CD11b (1:200; clone M1/70; BioLegend), CD11c (1:200; clone N418; BioLegend), and CD8a (1:200; clone 53-6.7; BioLegend). Tissue cells were then negatively enriched for CAFs, normal adjacent fibroblasts or CD4$^+$ T cells using iMag Streptavidin Particles Plus DM beads (BD, Cat# 557812), according to their protocol, using a DynaMag-2 magnetic system (Invitrogen).

imCAF or Treg cell sorting

Following negative enrichment with beads, cell suspensions were stained appropriate antibodies to isolate imCAFs, Treg cells, or CXCR3$^+$ Treg cells. For imCAF isolation, cells were blocked with 10% horse serum (Gibco, Cat# 16050122), washed, and incubated with mouse anti-CHL1 (1:200; clone MAB2126; R&D) in 2% horse serum at 4°C for 30 min. Cells were then washed twice and stained with mouse anti-rat AF647 secondary antibody (Invitrogen Cat# A48272) in 2% horse serum for 15 min on ice. Cells were then washed twice and stained with CD45.2-APC-Cy7 (1:200; clone 104; Tonbo), CD31-BV711 (1:400; clone 390; BioLegend), Epcam-PE-Dazzle594 (1:200; clone G8.8; BioLegend), Pdgfrα -BV605 (1:400; clone APA5; BioLegend), CD146-PE-Cy7 (1:400; clone ME-9F1; BioLegend). For Treg cell isolation experiments, we utilized male Foxp3^{GFP} mice. Cells were stained with PE-CXCR3 (1:200; clone CXCR3-173; BioLegend), CD8a-PerCP-Cy5.5 (1:400; clone 53-6.7; Tonbo), CD4-BV605 (1:400; clone RM4-5; BD Biosciences), CD44-APC-Cy7 (1:400; clone IM7; Tonbo), CD62L-APC (1:400; clone MEL-14; Tonbo). For live cell sorting, Sytox Blue (Thermofisher) was added directly to sample 3 min prior to sort to exclude dead cells. Fibroblasts were sorted using a SonyMA 900, and T cells were sorted using a BD FACSAria II.

Bone marrow chimera experiments

Recipient mice were irradiated twice before receiving donor bone marrow (2 x 5.25 Gy doses, 4h apart) for a total dose of 10.5 Gy (MultiRad350, Precision X-Ray). To extract donor bone marrow, femurs and tibias of donor mice were isolated and washed with EtOH (1 min). Bones were then ground in 5mL of HBSS (Gibco) with 10% FBS using a mortar and pestle under sterile conditions. Marrow was then isolated and strained through a 40μm cell strainer, and mortar was washed twice with 10mL of HBSS + FBS and ran though the same 40μm cell strainer. Marrow cells were pelleted, ACK lysed and were passed through a 40μm nylon mesh. To eliminate autoreactive T cells from $Foxp3^{KO/KO}$ bone marrow, we performed negative enrichment of $CD90.2^{+}$ cells prior to cell counting. Cells were then resuspended at $5x10^7$ cells/mL with serum-free HBSS. For mixed bone marrow chimera experiments, cells were then mixed to appropriate ratios.

For transfer of bone marrow, mice were anesthetized via isoflurane inhalation and injected retro-orbitally with 100μL of bone marrow ($5x10^6$ cells) immediately after the second round of radiation. Mice were then supplied with water supplemented with 0.5g/L vancomycin (Goldbio, Cat# V-200-1), 1g/L ampicillin (Goldbio, Cat# A-301-100) and 1g/L neomycin (Sigma, Cat# N6386-5G) for four weeks post bone marrow transfer, and reconstitution was checked via blood draw and flow cytometry analysis. Mice were used for tumor modeling experiments six weeks post bone marrow transfer.

Tumor volume measurements

Tumors from lungs were carefully dissected using forceps and dissection scissors under a stereoscope. Individual dimension measurements were made atop a ruler under a stereoscope. Individual tumor volumes were calculated using the ellipsoid formula $V = \frac{4}{3}\pi(length/2)(width/2)(height/2)$.

Cytotoxic killing assay with Treg cells

Splenocytes from an OT1 mouse were isolated and incubated with SIINFEKL peptide (0.5 μg/ml; Sigma Cat #S7951) and rh-IL-2 (100 U/ml; NIH) in RPMI 1640 supplemented with 5% FBS, 1% l-glutamine, 1% penicillin-streptomycin, 1% NEAA, 1% sodium pyruvate, 55μM BME, and 10 mM HEPES (pH 7.2-

7.5). On the same day, $CXCR3^-$ and $CXCR3^+$ Treg cells were FACS sorted from the spleens and lymph nodes of $FOXP3^{GFP}$ mice, and expanded utilizing Dynabeads Mouse T-Activator CD3/CD28 (Cat #11453D) according to manufacturer's instructions. After 5 days of activation/expansion, $CD8^+CD44^+CD62L^-$ T cells were FACS sorted and incubated with $LLC^{OVA\text{-}LUC}$ cells, plus $CXCR3^-$ or $CXCR3^+$ Treg cells, at a ratio of 1:1:4 ($LLC^{OVA\text{-}LUC}$:CD8:Treg). After 18h, cells were lysed and read on a luminometer per manufacturer's instructions (Promega #E1500). To calculate relative viability, luminescence readouts of experimental wells were divided by the luminescence of wells containing $LLC^{OVA\text{-}LUC}$ cells only minus the luminescence of the $LLC^{OVA\text{-}LUC}$ + CD8 only "max lysis" control.
