# Supplementary material for: A novel CAF population coordinates hyper-suppressive regulatory T cell recruitment and localization in lung cancer
Source: Nat Immunol. 2026 Aug 11;27(9):1899–912. doi: 10.1038/s41590-026-02607-2 (PMC13506330; doi:10.1038/s41590-026-02607-2)
Supplement: Supplementary file 2 [file 41590_2026_2607_MOESM2_ESM.pdf]

nature portfolio

Corresponding author(s): Nicholas Arpaia

Last updated by author(s): Jul 8, 2026

Reporting Summary

Nature Portfolio wishes to improve the reproducibility of the work that we publish. This form provides structure for consistency and transparency in reporting. For further information on Nature Portfolio policies, see our Editorial Policies and the Editorial Policy Checklist.

Statistics

For all statistical analyses, confirm that the following items are present in the figure legend, table legend, main text, or Methods section.

n/a	Confirmed	
☐	☒	The exact sample size (*n*) for each experimental group/condition, given as a discrete number and unit of measurement
☐	☒	A statement on whether measurements were taken from distinct samples or whether the same sample was measured repeatedly
☐	☒	The statistical test(s) used AND whether they are one- or two-sided<br>*Only common tests should be described solely by name; describe more complex techniques in the Methods section.*
☐	☒	A description of all covariates tested
☐	☒	A description of any assumptions or corrections, such as tests of normality and adjustment for multiple comparisons
☐	☒	A full description of the statistical parameters including central tendency (e.g. means) or other basic estimates (e.g. regression coefficient) AND variation (e.g. standard deviation) or associated estimates of uncertainty (e.g. confidence intervals)
☐	☒	For null hypothesis testing, the test statistic (e.g. *F*, *t*, *r*) with confidence intervals, effect sizes, degrees of freedom and *P* value noted<br>*Give P values as exact values whenever suitable.*
☐	☒	For Bayesian analysis, information on the choice of priors and Markov chain Monte Carlo settings
☐	☒	For hierarchical and complex designs, identification of the appropriate level for tests and full reporting of outcomes
☒	☐	Estimates of effect sizes (e.g. Cohen's *d*, Pearson's *r*), indicating how they were calculated

Our web collection on statistics for biologists contains articles on many of the points above.

Software and code

Policy information about availability of computer code

Data collection	No original code was used in this study for data collection.
Data analysis	No original code was used in this study for data collection. Software used for data analysis in this study includes bclfastq2 (version 2.19) kallisto (version 0.44.0), Python (version 3.9), Space Ranger (version 2.1), Cell Ranger (version 7.1), scanpy (version 1.10), squidpy (version 1.6.2), QuPath (version 0.3.0), FlowJo (version 10.7.1), Fiji (version 1), R (version 4.2.2), Prism (version 10.0) Microsoft Excel (version 16.99), Microsoft Word (version 16.99)

For manuscripts utilizing custom algorithms or software that are central to the research but not yet described in published literature, software must be made available to editors and reviewers. We strongly encourage code deposition in a community repository (e.g. GitHub). See the Nature Portfolio guidelines for submitting code & software for further information.

Data

Policy information about availability of data

All manuscripts must include a data availability statement. This statement should provide the following information, where applicable:

- Accession codes, unique identifiers, or web links for publicly available datasets
- A description of any restrictions on data availability
- For clinical datasets or third party data, please ensure that the statement adheres to our policy

RNA-seq, scRNA-seq, and spatial transcriptomics data generated in this study have been deposited in NCBI's Gene Expression Omnibus under GEO accession number GSE303166. All other data generated in this study are provided in the Supplementary Information.

Research involving human participants, their data, or biological material

Policy information about studies with human participants or human data. See also policy information about sex, gender (identity/presentation), and sexual orientation and race, ethnicity and racism.

Reporting on sex and gender	Prior to surgery, all patients had given surgical informed consent, which was approved by the Columbia University Institutional Review Board. (AAAB2667). Lung tumor tissue was retrieved from the Columbia University Irving Medical Center Database Shared Resources (DBSR)/Columbia University Tumor Bank. Samples were deidentified prior to receipt in accordance with our Institutional Review Board protocol (AAAB2667).
Reporting on race, ethnicity, or other socially relevant groupings	As above (AAAB2667).
Population characteristics	As above (AAAB2667).
Recruitment	As above (AAAB2667).
Ethics oversight	As above (AAAB2667).

Note that full information on the approval of the study protocol must also be provided in the manuscript.

Field-specific reporting

Please select the one below that is the best fit for your research. If you are not sure, read the appropriate sections before making your selection.

☒ Life sciences ☐ Behavioural & social sciences ☐ Ecological, evolutionary & environmental sciences

For a reference copy of the document with all sections, see nature.com/documents/nr-reporting-summary-flat.pdf

Life sciences study design

All studies must disclose on these points even when the disclosure is negative.

Sample size	As determined by previous studies, 2-3 separate experiments were performed in experiments involving western blotting. As determined by previous studies, in vivo experimentation was done using 3 separate cohorts of mice (~5 mice per group). For single cell RNA sequencing and bulk sequencing experiments, cells from 3 mice were used for each sequencing run. For spatial transcriptomics, lung sections from 2 individual murine lungs bearing tumors were sectioned on to two separate Visium slides.
Data exclusions	As detailed in Methods, human lung tumor tissue samples received were included except for one, which was comprised of predominantly (>95%) mucin and scant neoplastic epithelium
Replication	All attempts at replication were successful.
Randomization	Samples for in vivo experiments were sorted in experimental groups based on genotype, with order of experimentation randomly determined by genotyping order. Each cage included mice from both genotypes, to control for any microbiota differences between cages.
Blinding	No measurements in this study relied on metrics susceptible to bias due to blinding status; therefore, blinding was not necessary for the assessment of experimental or disease-related parameters

Reporting for specific materials, systems and methods

We require information from authors about some types of materials, experimental systems and methods used in many studies. Here, indicate whether each material, system or method listed is relevant to your study. If you are not sure if a list item applies to your research, read the appropriate section before selecting a response.

Materials & experimental systems

n/a	Involved in the study
☐	☒ Antibodies
☐	☒ Eukaryotic cell lines
☒	☐ Palaeontology and archaeology
☐	☒ Animals and other organisms
☒	☐ Clinical data
☒	☐ Dual use research of concern
☒	☐ Plants

Methods

n/a	Involved in the study
☒	☐ ChIP-seq
☐	☒ Flow cytometry
☒	☐ MRI-based neuroimaging

Antibodies

Antibodies used

Human lung tissue histological staining antibodies: CHL1 (Abcam, # ab106269) and FOXP3 (Invitrogen; clone 236A/E7; # 14477782). Murine lung tissue immunofluorescence staining: anti-CD4 (Abcam, #ab183685) and rat anti-Foxp3 (Invitrogen, #14577382), rabbit anti-CD8 (Abcam, #ab217344), rabbit anti-GFP (Invitrogen, #A21311), Syrian hamster anti-podoplanin (AngioBio, #11-033), goat anti-CHL1 (R&D, #AF2147), rat anti-CHL1 (R&D, #MAB2147), rat anti-CXCL9 (R&D, #AF-492), donkey anti-rat AF488, donkey anti-rabbit Cy5, donkey anti-goat Cy5, donkey anti-goat Cy3, donkey anti-rat Cy3, goat anti-syrian hamster Cy5. (Jackson Immunoresearch), rat anti-Tenasin C (.R&D, Cat# MAB2138), mouse anti-smooth muscle actin (Sigma, Cat# C6198). HTOs and flow cytometry antibodies:
TotalSeq™-B0301 anti-mouse Hashtag 1 Antibody (BioLegend, Cat# 155831, RRID:AB_2814067),
TotalSeq™-B0302 anti-mouse Hashtag 2 Antibody (BioLegend, Cat# 155833, RRID:AB_2814068),
TotalSeq™-B0303 anti-mouse Hashtag 3 Antibody (BioLegend, Cat# 155835, RRID:AB_2814069),
TotalSeq™-B0304 anti-mouse Hashtag 4 Antibody (BioLegend, Cat# 155837, RRID:AB_2814070),
TotalSeq™-B0305 anti-mouse Hashtag 5 Antibody (BioLegend, Cat# 155839, RRID:AB_2814071),
TotalSeq™-B0306 anti-mouse Hashtag 6 Antibody (BioLegend, Cat# 155841, RRID:AB_2814072),
Biotin anti-mouse CD326 (Ep-CAM) (BioLegend, Cat# 118204, RRID:AB_1134178),
Biotin anti-mouse CD19 (BioLegend, Cat# 152420, RRID:AB_2936614),
Biotin anti-mouse NK1.1 (BioLegend, Cat# 108704, RRID:AB_313391),
Biotin anti-mouse Ter-119 (BioLegend, Cat# 116204, RRID:AB_313705),
Biotin anti-mouse CD45 (BioLegend, Cat# 103104, RRID:AB_312969),
Biotin anti-mouse CD31 (BioLegend, Cat# 102404, RRID:AB_312899),
Biotin anti-mouse CD8a (BioLegend, Cat# 100704, RRID:AB_312743),
Biotin anti-mouse CD140a (BioLegend, Cat# 135910, RRID:AB_2043974),
Biotin anti-mouse CD11b (BioLegend, Cat# 101204, RRID:AB_312787),
Biotin anti-mouse CD11c (BioLegend, Cat# 117304, RRID:AB_313773),
Purified Anti-Mouse CD16 / CD32 (Fc Shield) (Cytek, Cat# 70-0161, RRID:AB_2621487),
APC-Cyanine7 Anti-Mouse CD45.2 (Cytek, Cat# 25-0454, RRID:AB_3677430),
Brilliant Violet 711 anti-mouse CD31 (BioLegend, Cat# 102449, RRID:AB_2860594),
PE/Dazzle 594 anti-mouse CD326 (Ep-CAM) (BioLegend, Cat# 118236, RRID:AB_2632777),
Brilliant Violet 605 anti-mouse CD140a (BioLegend, Cat# 135916, RRID:AB_2721548),
PE/Cyanine7 anti-mouse CD146 (BioLegend, Cat# 134714, RRID:AB_2563109),
PE anti-mouse CD183 (CXCR3) (BioLegend, Cat# 155904, RRID:AB_2783131),
PerCP/Cyanine5.5 anti-mouse CD8a (Cytek, Cat# 65-0081, RRID:AB_2621882),
BV605 Rat Anti-Mouse CD4 (BD Biosciences, Cat# 563151, RRID:AB_2687549),
PE-Cyanine7 Anti-Human/Mouse CD44 (Tonbo Biosciences, Cat# 60-0441, RRID:AB_2621847),
APC Anti-Mouse CD62L (Tonbo Biosciences, Cat# 20-0621, RRID:AB_2621582),
PE Anti-Mouse CD8α (Tonbo Biosciences, Cat# 50-0081, RRID:AB_2621741),
FITC Anti-Mouse/Rat FOXP3 (Thermo Fisher Scientific, Cat# 11-5773-82, RRID:AB_465243),
BV711 Hamster Anti-Mouse TCRβ (BD Biosciences),
Anti-Ki-67 Alexa Fluor 700 (Fisher Scientific/Invitrogen, Cat# 50-112-4690, RRID:AB_2688057),
BUV395 Rat Anti-Mouse CD45 (BD Biosciences, Cat# 564279, RRID:AB_2651134),
BUV496 Hamster Anti-Mouse CD3e (BD Biosciences, Cat# 612955, RRID:AB_2870231),
BV786 Hamster Anti-Mouse CD69 (BD Biosciences, Cat# 564683, RRID:AB_2738890),
APC Anti-Mouse KLRG1 (Tonbo Biosciences, Cat# 20-5893, RRID:AB_2621607),
PE/Cyanine7 anti-mouse CD279 (PD-1) (BioLegend, Cat# 135216, RRID:AB_10689635),
CD152 (CTLA-4) Monoclonal Antibody, APC (Thermo Fisher Scientific, Cat# 17-1522-82, RRID:AB_2016700),
CD357 (AITR/GITR) Monoclonal Antibody, PE-Cyanine7 (Thermo Fisher Scientific, Cat# 25-5874-82, RRID:AB_10548516),
Brilliant Violet 650 anti-mouse/human CD44 (BioLegend, Cat# 103049, RRID:AB_2562600),
BUV737 Rat Anti-Mouse CD4 (BD Biosciences, Cat# 612844, RRID:AB_2870166),
PE/Dazzle 594 anti-mouse CD8a (BioLegend, Cat# 100762, RRID:AB_2564027),
violetFluor 450 Anti-Mouse CD45.1 (Tonbo Biosciences, Cat# 75-0453, RRID:AB_2621949),
PerCP-Cyanine5.5 Anti-Mouse CD45.2 (Tonbo Biosciences, Cat# 65-0454, RRID:AB_2621894),

Validation

Validation of each antibody was done by the companies (included in the product descriptions for each antibody).

Eukaryotic cell lines

Policy information about cell lines and Sex and Gender in Research

Cell line source(s)	LLC (also known as LL/2 or LLC1): ATCC (Catalog # CRL-1642); B16F10 and KPCy: Robert Schwabe at Columbia University Irving Medical Center
Authentication	None of the cell lines used were authenticated.
Mycoplasma contamination	All cell lines tested negative for mycoplasma contamination.
Commonly misidentified lines (See ICLAC register)	None

Animals and other research organisms

Policy information about studies involving animals; ARRIVE guidelines recommended for reporting animal research, and Sex and Gender in Research

Laboratory animals	Mus musculus, C57BL/6, 8-12 weeks old
Wild animals	No wild animals were used in this study
Reporting on sex	For all experiments, females were used unless otherwise indicated in the manuscript (Foxp3KO bone marrow chimera, Cxcr3KO bone marrow chimera)
Field-collected samples	This study did not involved collection of samples from the field
Ethics oversight	Columbia University Institutional Animal Care and Use Committee (protocol #AC-AABT2656)

Note that full information on the approval of the study protocol must also be provided in the manuscript.

Plants

Seed stocks	Not applicable, no plants were used in this study.
Novel plant genotypes	Not applicable, no plants were used in this study.
Authentication	Not applicable, no plants were used in this study.

Flow Cytometry

Plots

Confirm that:

- [x] The axis labels state the marker and fluorochrome used (e.g. CD4-FITC).
- [x] The axis scales are clearly visible. Include numbers along axes only for bottom left plot of group (a 'group' is an analysis of identical markers).
- [x] All plots are contour plots with outliers or pseudocolor plots.
- [x] A numerical value for number of cells or percentage (with statistics) is provided.

Methodology

Sample preparation	See Methods and Supplemental Methods
Instrument	BD FACSAria II, BD LSR Fortessa, Sony MA900
Software	FlowJo, BD FACSDiva
Cell population abundance	Cell abundances were quantified as percentages based on gating strategies. Post-sort purity was assessed by re-analyzing

Cell population abundance	sorted populations on the flow cytometer to confirm that the majority of collected cells fell within the intended collection gates.
Gating strategy	See Supplementary Figures/Methods

☒ Tick this box to confirm that a figure exemplifying the gating strategy is provided in the Supplementary Information.
